# Chest imaging using signs, symbols, and naturalistic images: a practical guide for radiologists and non-radiologists

**DOI:** 10.1186/s13244-019-0789-4

**Published:** 2019-12-04

**Authors:** Alessandra Chiarenza, Luca Esposto Ultimo, Daniele Falsaperla, Mario Travali, Pietro Valerio Foti, Sebastiano Emanuele Torrisi, Matteo Schisano, Letizia Antonella Mauro, Gianluca Sambataro, Antonio Basile, Carlo Vancheri, Stefano Palmucci

**Affiliations:** 1 Department of Medical Surgical Sciences and Advanced Technologies “GF Ingrassia” – Radiology Unit I, University Hospital “Policlinico-Vittorio Emanuele”, 95123 Catania, Italy; 2 Regional Referral Center for Rare Lung Disease, University Hospital Policlinico-Vittorio Emanuele, Department of Clinical and Experimental Medicine, University of Catania, Catania, Italy; 3 Center for interstitial and rare lung diseases, Pneumology, Thoraxklinik, University of Heidelberg, Germany and German Center for Lung Research, Heidelberg, Germany; 4 Artroreuma S.R.L. – Rheumatology Outpatient Clinic accredited with the Italian National Health System, Mascalucia, Catania, Italy

**Keywords:** Thorax, Multidetector computed tomography, Thoracic diseases

## Abstract

Several imaging findings of thoracic diseases have been referred—on chest radiographs or CT scans—to signs, symbols, or naturalistic images. Most of these imaging findings include the air bronchogram sign, the air crescent sign, the arcade-like sign, the atoll sign, the cheerios sign, the crazy paving appearance, the comet-tail sign, the darkus bronchus sign, the doughnut sign, the pattern of eggshell calcifications, the feeding vessel sign, the finger-in-gloove sign, the galaxy sign, the ginkgo leaf sign, the Golden-S sign, the halo sign, the headcheese sign, the honeycombing appearance, the interface sign, the knuckle sign, the monod sign, the mosaic attenuation, the Oreo-cookie sign, the polo-mint sign, the presence of popcorn calcifications, the positive bronchus sign, the railway track appearance, the scimitar sign, the signet ring sign, the snowstorm sign, the sunburst sign, the tree-in-bud distribution, and the tram truck line appearance. These associations are very helpful for radiologists and non-radiologists and increase learning and assimilation of concepts.

Therefore, the aim of this pictorial review is to highlight the main thoracic imaging findings that may be associated with signs, symbols, or naturalistic images: an “iconographic” glossary of terms used for thoracic imaging is reproduced—placing side by side radiological features and naturalistic figures, symbols, and schematic drawings.

## Key points

- In thoracic imaging, some terms refer to symbols or naturalistic images.
- Associations provide clear and quick explanation of the associated radiological pattern.
- The reference to symbols and/or naturalistic images provides tips and tricks

## Background

In 2008, the Fleischner Society listed imaging terms used for description of main thoracic radiological signs [1]. This classification updated previous glossaries published in 1984 and 1996 [2, 3]. The glossary paper offers some radiological examples of these terms, providing a useful guide for radiologists. In addition, some terms listed in the glossary paper—such as the atoll sign or crazy paving sign—clearly refer to symbols or naturalistic images—and these associations provide a clear and quick explanation of the associated radiological pattern.

The human brain has different types of learning mechanisms, based on personal ways of sensing, elaborating, and retrieving information from memory. In this regard, the association with symbols and photos represents a series of tips and tricks to increase learning and assimilation of concepts. Therefore, visual memory and visual-iconographic learning have been introduced into radiological language—in the majority of cases creating an association between CT and X-ray findings depicted in images and symbols of nature. Using these associations, our brain creates an unconscious emotion linked to images, which makes the memory stronger and simplifies the learning process. In addition, the knowledge of these radiological signs increases the diagnosis specificity, there being a strong association between these imaging features and certain thoracic diseases.

Therefore, the aim of this pictorial review is to propose an iconographic version of the glossary terms of thoracic radiological findings. Each radiological finding—from A to Z—is described providing an association with symbols, naturalistic figures and photos, and schematic drawings.

## Main text

### Air bronchogram

**Fig. 1:**
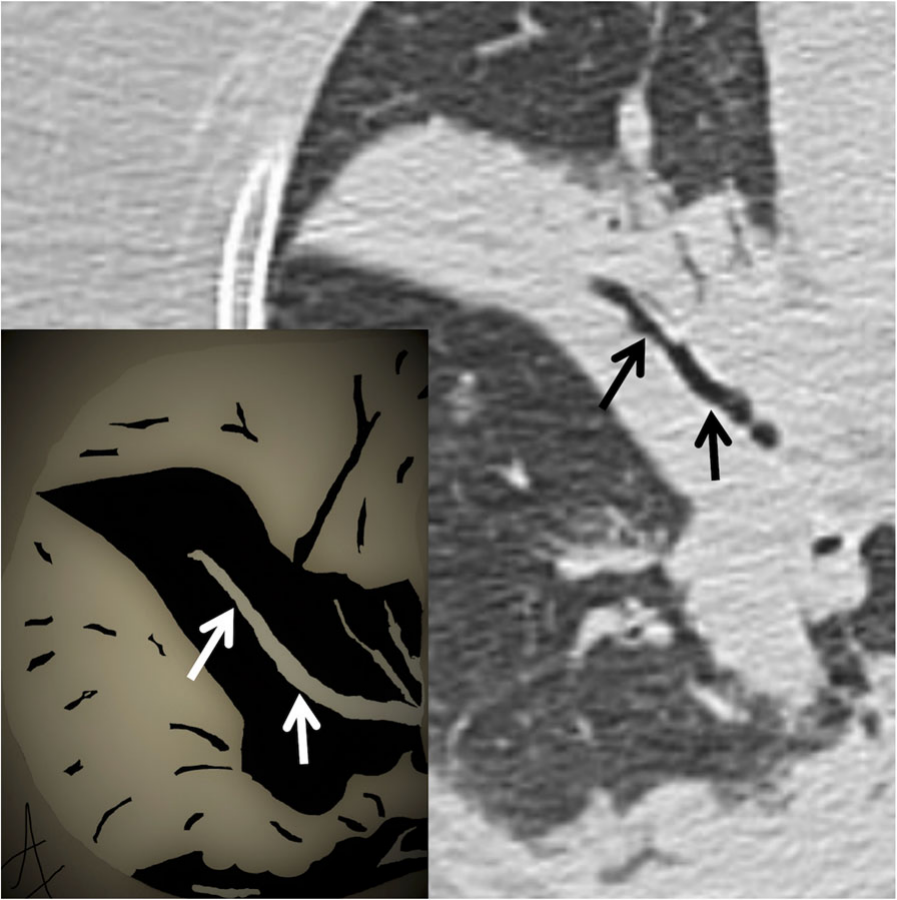
Air bronchogram sign. The figure clearly shows patent bronchus (black arrows) inside a pulmonary consolidation, in a patient with pneumonia. The embedded drawing demonstrates the correspondent patent bronchus inside the consolidation (white arrows)

According to the Fleischner Society glossary, the air bronchogram sign refers to the visualization of bronchial structures containing air—strongly hypodense on CT images—in the context of consolidation areas of the surrounding pulmonary parenchyma (Fig. 1) [1]. It is a pivotal sign of pulmonary semeiotics, reported in literature as early as 1969 by Remy J [4]. Numerous pathological processes that fill alveolar air spaces—such as pneumonia, edema, infarction, pulmonary hemorrhages, aspirations, traumas—may reproduce the air bronchogram sign, which is frequently recognizable on both chest radiographs and CT images. Pulmonary edema is a pathological process with cardiac dysfunction and accumulation of fluids at the interstitial and alveolar level and therefore with the possibility of producing the air bronchogram sign [5]. In the case of edema from ARDS (acute respiratory distress syndrome), the air bronchogram sign is not associated with cardiomegaly; the distribution of the edemigenic consolidations is widespread, patchy, with a tendency to peripheral localization. In non-obstructive pulmonary atelectasis, the air bronchogram sign is frequently located at lower lobes and could be associated with a decrease in pulmonary volume. In cases of unsolved infectious consolidations showing air bronchogram, namely after several weeks of antibiotic therapy, a suspicion of pulmonary adenocarcinoma with lepidic pattern or pulmonary lymphoma may be considered [5, 6].

### Air crescent sign

**Fig. 2:**
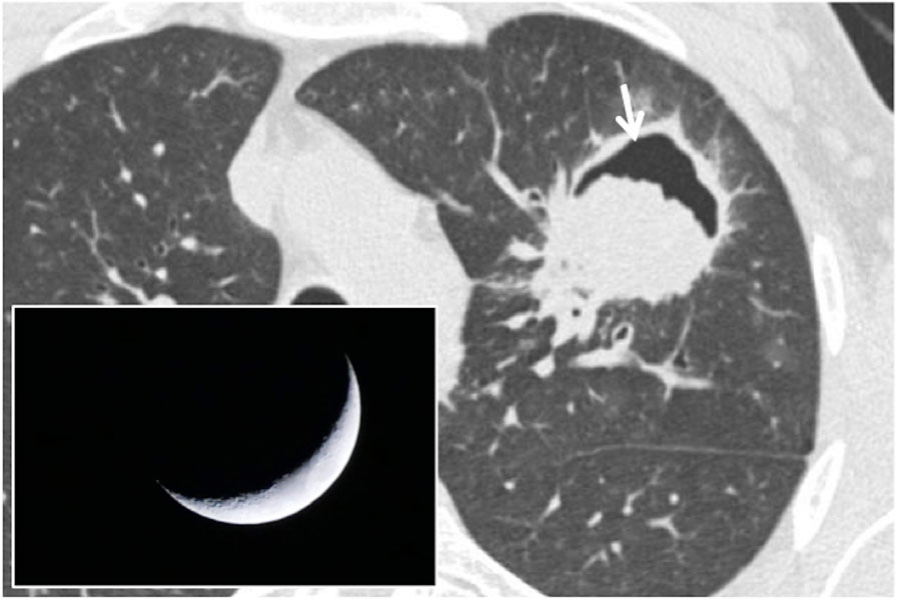
Air crescent sign. CT scan of a patient with pulmonary aspergillosis showing a necrotic and cavitated lesion (white arrow) in left upper lobe. The air filling the cavitation looks like the shadow of a crescent moon—which is demonstrated in the embedded figure

The air crescent sign is a radiological sign, better appreciable on multidetector computed tomography (MDCT) studies, characterized by the appearance of air in invasive and semi-invasive aspergillosis lesions (Fig. 2) [7]. The first description, testifying that the sign is not pathognomonic of an infectious aspergillosis lesion, dates back to 1975, when Bard and Hassani described “the appearance of a crescent sign in a pulmonary hematoma”; however, the authors reported that the presence of this finding in a “coin-lesion” is not specific [8]. In 1979, Curtis et al. described this sign for an invasive form of aspergillosis [9]. The aspergillus is a microorganism that initially proliferates at endobronchial level and subsequently invades the bloodstream through the bronchial arteries, with thrombosis; the obliteration of the vascular lumen involves the onset of necrosis and cavitation in the context of the lesions. In case of invasive aspergillosis, MDCT images demonstrate areas of increased density with peripheral ground glass or thickenings and/or multiple peribronchial nodules with irregular profile; 2 weeks after their appearance, these areas of increased density undergo necrosis and cavitation, with subsequent formation of the air crescent sign (after about 20 days). The appearance of this sign may be explained by the peripheral reabsorption of the necrotic tissue developed in the central portion: the residual part is replaced by air. The air crescent sign can be visualized in 50% of patients affected by invasive aspergillosis [10]. The reabsorption of the necrotic area coincides with a prognostic improvement and a progressive recovery from infection [11].

Several authors reported that air crescent sign could be found in necrotic areas of the pulmonary parenchyma which arise from other causes—such as tuberculosis, abscesses, carcinomas, or parasitic lesions (hydatidosis) [7, 8].

### Arcade-like sign

**Fig. 3:**
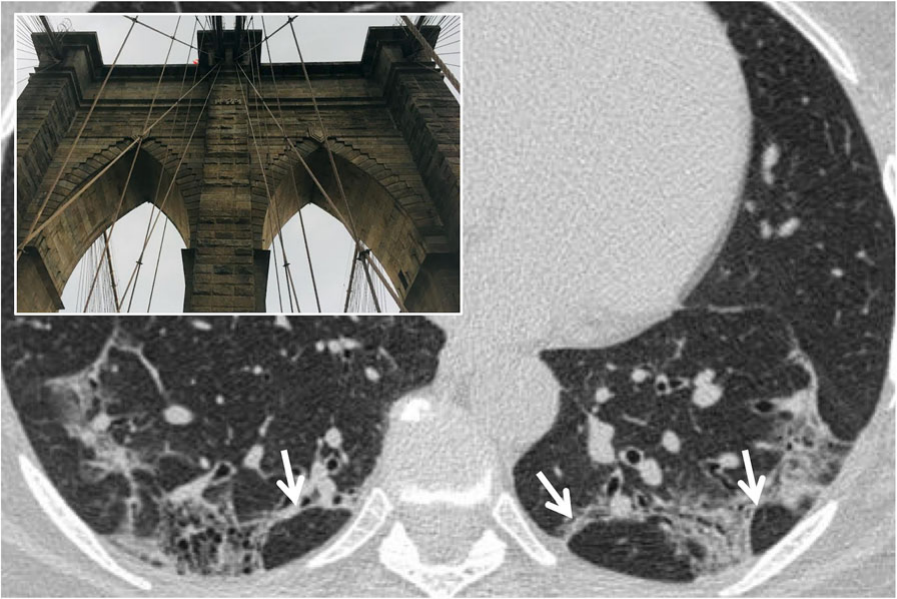
Arcade-like sign. Perilobular fibrosis in a patient with COP disease, which appears as curved or arched consolidation bands, with shaded margins, distributed around the structures surrounding the secondary pulmonary lobules (white arrows). This pattern resembles an arcade appearance (as reproduced in the embedded picture)

It refers to the typical feature of perilobular fibrosis frequently found in COP (cryptogenic organizing pneumonia). In 2004, Ujitaet et al. [12] found the presence of perilobular fibrosis, with an “arch” pattern, in more than half of the patients with COP; this sign may be also related to the presence of perilobular inflammation. It shows itself in the form of curved or arched consolidation bands, with shaded margins, distributed around the structures surrounding the secondary pulmonary lobules (Fig. 3); it often reaches the pleural surface [12].

### Atoll sign

**Fig. 4:**
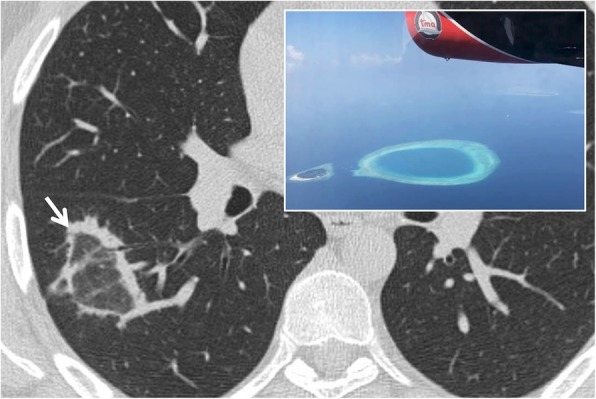
Atoll sign. The so called “atoll sign” (white arrow) is represented by a central ground glass area bordered by a peripheral consolidation. This CT finding—similar to the oceanic atoll—is frequently observed in patients with COP; it has been also called “reversed halo sign”

The atoll sign, also called “reversed halo sign”, is described as an area of increased density of ground glass in the center, delimited for at least three quarters by an area of parenchymal consolidation [13], with a thickness of at least 2 mm (Fig. 4). The term atoll was reported for the first time in literature in a case report in 1999 by Zompatori et al. [13]; in this article, the alteration of BOOP (bronchiolitis obliterans organizing pneumonia), with an appearance of ring-shaped opacity, was defined as “sign of the atoll.” The pathogenesis of this sign derives from the inflammation of the alveolar septa and from cellular debris in the alveoli, thus describing the central ground glass and granulation tissue of the circumferential thickening. Initially, it was described as highly specific for COP and BOOP: in 1996, ground glass areas surrounded by consolidation zones were described by Voloudakiet et al. as “crescentic and ring-shaped opacities” [14]. Subsequently, ring-shaped opacities with an atoll appearance have been associated with many other pulmonary diseases [15], such as atypical sarcoidosis, granulomatosis with polyangiitis, *Pneumocystis jirovecii* pneumonia, adenocarcinomas, pulmonary infarctions, or outcomes of radiation therapy (in the first 4–12 weeks post-therapy) [16, 17]. More in detail, Marchiori et al. have reported the presence of the atoll sign in infectious and non-infectious pulmonary diseases: among infective conditions, the reverse halo sign may be observed in cases of paracoccidioidomycosis or zygomycosis [15]. It may be also related to invasive fungal opportunistic infections—especially in immunocompromised patients, with conditions such as leukemia and graft-vs-host disease; Legouge et al. recently (2014) described the presence of a “reversed halo sign” in a case of pulmonary mucormycosis, arising in a neutropenic leukemic patient [18]. The atoll sign—when associated with other centrilobular nodules—may also be an expression of active pulmonary tuberculosis [6, 19]; in cases of active granulomatous diseases, the ring of the reverse halo sign may reproduce a nodular appearance [15].

Among non-infectious conditions, it has been depicted in cases of COP, Wegener granulomatosis, lymphomatoid granulomatosis, and sarcoidosis; due to the wide spectrum of pulmonary diseases that may reproduce this CT features, the atoll sign represents a non-specific sign [15].

Recently, nodular and reticular imaging patterns have been described for the reverse halo sign in another article by Marchiori et al. [20]. The nodular pattern refers to the presence of nodules on the border or within the reverse halo sign; this imaging appearance has been associated with granulomatous disorders (tuberculosis and sarcoidosis). The reticular pattern is characterized by reticulations within the reverse halo sign: this condition may be associated with invasive fungal diseases (immunocompromised patients) or pulmonary infarctions (immunocompetent patients) [20].

### Cheerios sign

**Fig. 5:**
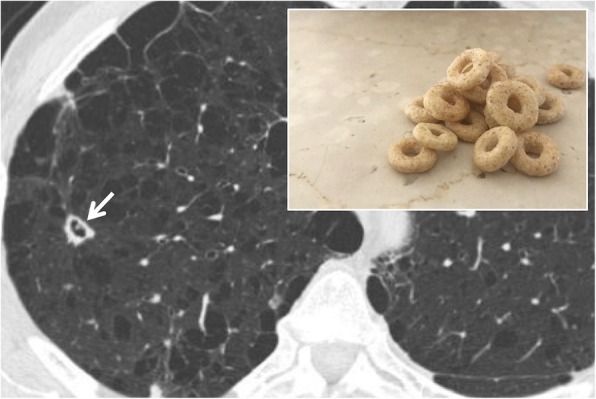
Cheerios sign. The cheerios sign is due to cell proliferation around a bronchial branch (white arrow); it may be found in patients with Langerhans Cell histiocytosis or lung adenocarcinoma. It is very similar to the appearance of the famous breakfast cereal—as well demonstrated in the embedded figure

The cheerios sign is a sign depicted in axial tomographic images: it consists of pulmonary nodules containing a small central air cavity, supplied by a patent bronchus (Fig. 5). It is produced by cell proliferation, malignant or not, around an un-occluded bronchial branch. It was described in 1993 by Reed and O’Neil [21]; the sign was referred to the onset of low-grade pulmonary adenocarcinomas. Histologically, tumors that can reproduce cheerios sign are adenocarcinoma in situ, minimally invasive adenocarcinoma, invasive adenocarcinoma with a predominantly lepidic component, or invasive mucinous adenocarcinoma. In 1997, Lee et al. associated this sign with the presence of nodular lesions from pulmonary adenocarcinoma with lepidic pattern [22]. It can also be associated with Langerhans’ X histiocytosis or with meningothelial pulmonary nodules [23]. The nodules that reproduce the cheerios appearance should be distinguished by cavitated nodules—in which the excavation area is due to the necrotic phenomena—and not to the proliferation of tissue around an airway.

### Crazy paving

**Fig. 6:**
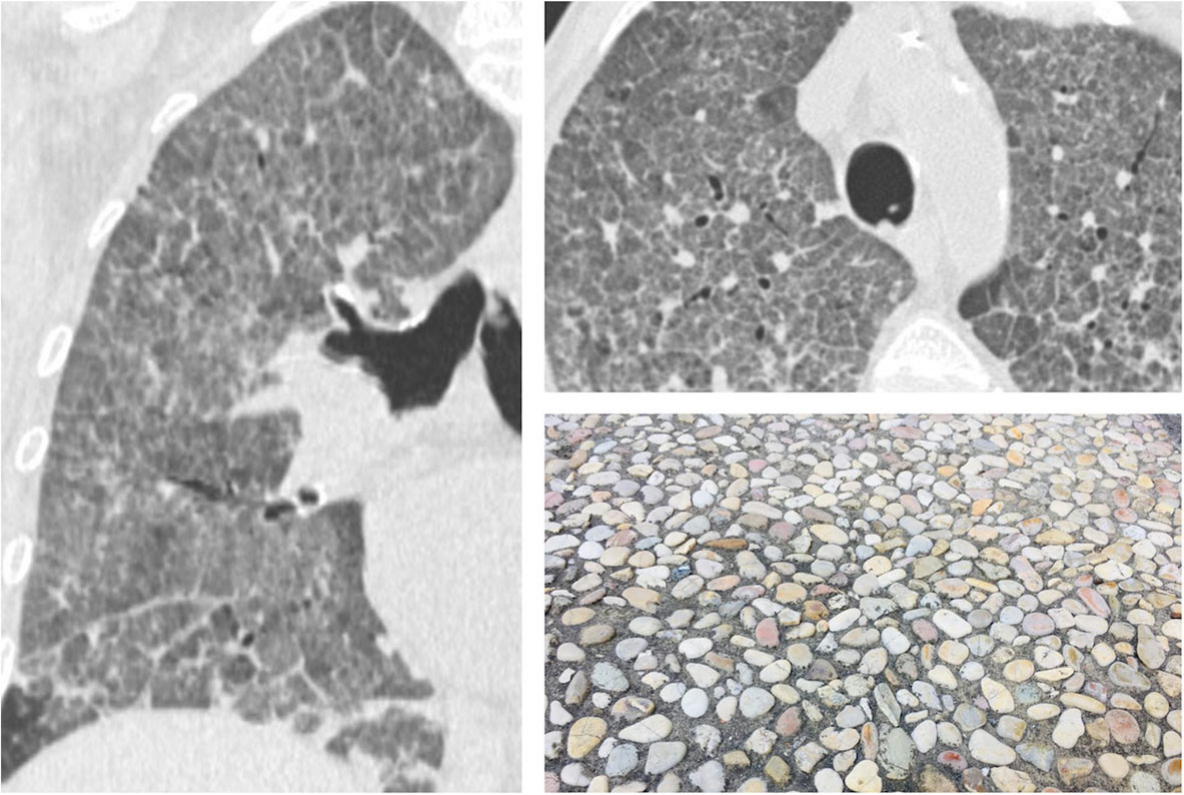
Crazy paving. Coronal (left side) and axial (right side) CT images show ground glass attenuation of the lungs—with superimposed reticulations, in a patient with *Pneumocystis jirovecii* infection. This appearance looks like the “roman crazy paving” and may be associated also with different pulmonary diseases (alveolar proteinosis, ARDS, drug-induced pneumonitis, etc.)

The “crazy paving” is a nonspecific pulmonary appearance, caused by increased density of lung parenchyma, with a ground glass appearance, superimposed on a reticular thickening of the inter- and intra-lobular septa (Fig. 6) [6, 24, 25]. This sign was originally recognized in patients with pulmonary proteinosis, a rare pathology with alveolar filling by proteinaceous material rich in lipids, associated with inflammation of the interstitium which reproduces the secondary pulmonary lobule with polygonal shapes. Other causes are represented by bacterial pneumonia, *Pneumocystis jirovecii*, drug-induced diseases, and pulmonary adenocarcinoma with lepidic pattern; in addition, the crazy paving appearance may be also associated with interstitial diseases—NSIP (nonspecific interstitial pneumonia) or COP, sarcoidosis, and many causes of pulmonary hemorrhage—such as granulomatosis with polyangiitis and Goodpasture’s syndrome [26].

### Comet tail sign

**Fig. 7:**
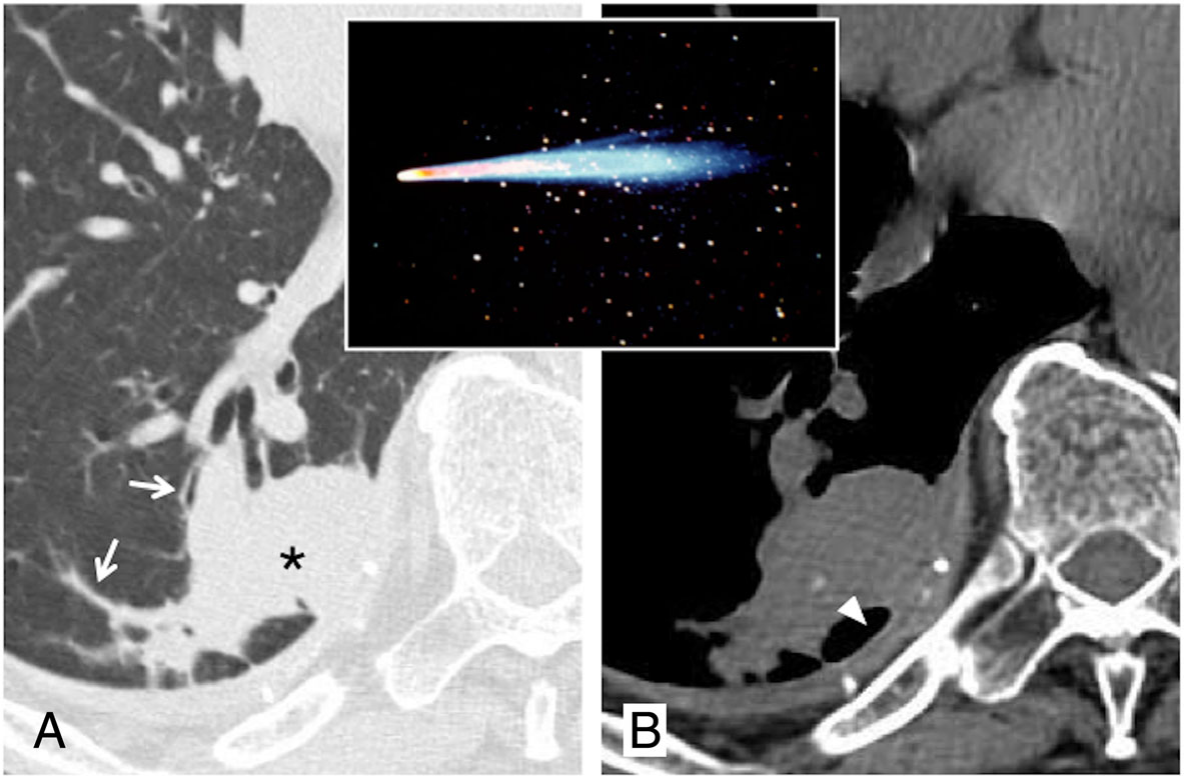
Comet tail sign. Axial CT image (**a**) shows a round consolidation (black asterisk) and stretched vessel and bronchus (white arrows): these combinations—reproducing a “comet tail” at the border of a round lesion—suggest the presence of round atelectasis. On axial CT image (**b**, mediastinal window), a dorsal pleural plaque with calcification is also appreciable (white arrowhead)

The “comet tail” sign consists in a curvilinear opacity that leads from a subpleural mass towards the ipsilateral hilum (Fig. 7). It was described in 1986 in a case report by Verschakelen [27]. The “tail of the comet” represents the distorted vessels and bronchus near the contiguous area of the round atelectasis. Frequently, a thickening of the adjacent pleura and a displacement of the adjacent fissures are associated. Round atelectasis is a form of rare parenchymal collapse which occurs adjacent to the pleural surface, with a frequent presence of air bronchogram in the context. Over time, it has been given various names, such as “Blesovsky syndrome” or “atelectasic pseudotumor,” often reproducing a tumor-like appearance. The atelectasis may be explained by the presence of irritant substances along the pleural surface, such as asbestos: consequently, the pleura appears thickened, and the pulmonary parenchyma may be contracted—developing a round form of atelectasis [6, 28].

### Dark bronchus sign

**Fig. 8:**
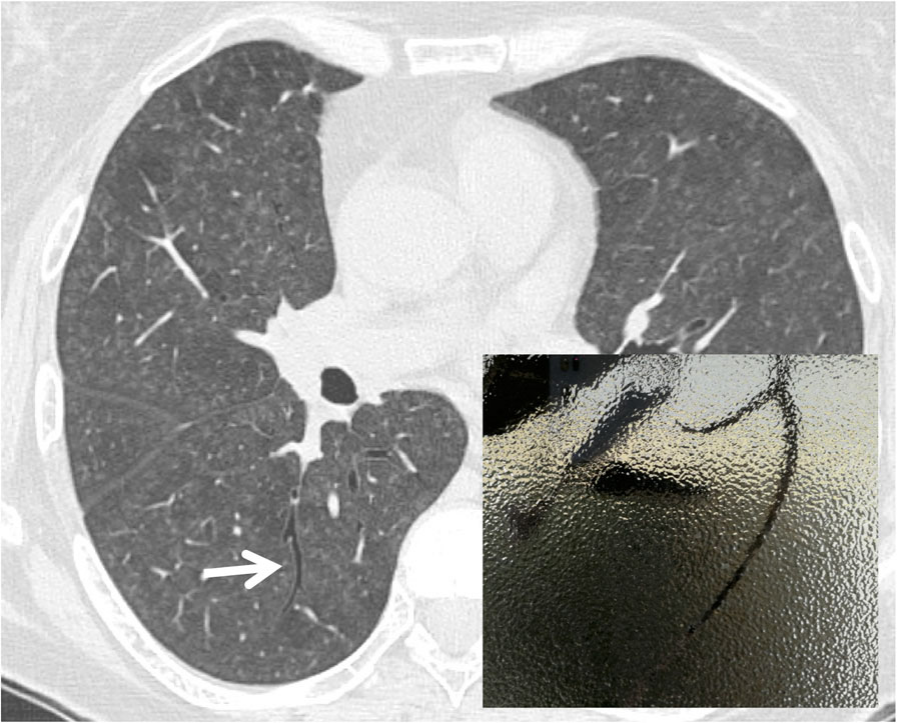
Dark bronchus sign. The figure shows a patent bronchial structure (white arrow)—which appears slightly dark—in the context of lung parenchyma area with a ground glass appearance. The dark bronchus sign is very useful to recognize pulmonary infection by *Pneumocystis jirovecii*

The “dark bronchus sign” is a sign consisting in the visualization of an apparently darker bronchus in the context of lung parenchyma area with a ground glass appearance (Fig. 8). This sign should be distinguished from the “air bronchogram,” which instead is represented by the visualization of a bronchial structure pervading a zone of pulmonary consolidation. In 2007, Yadav et al. [29] described the case of a 50-year-old HIV-infected man, with *Pneumocystis jiroveci* pneumonia: on MDCT images, a darker than normal bronchus was reported by authors; this appearance was related to an increased density of adjacent lung [29]. Pneumocystis infection is characterized by ground glass areas, with “patchy” or diffuse distribution; centrilobular nodules, pleural effusions, and lymphadenomegalies may occur. In some cases, the increase in lung density is very slight and assumes widespread distribution: this symmetrical, slight, and uniform ground glass appearance may be difficult to recognize—especially if it is diffused, with no recognizable areas of healthy parenchyma to be compared to. In these circumstances, the dark bronchus sign is very useful in recognizing pulmonary infection by *Pneumocystis jirovecii* [29].

### Doughnut sign

**Fig. 9:**
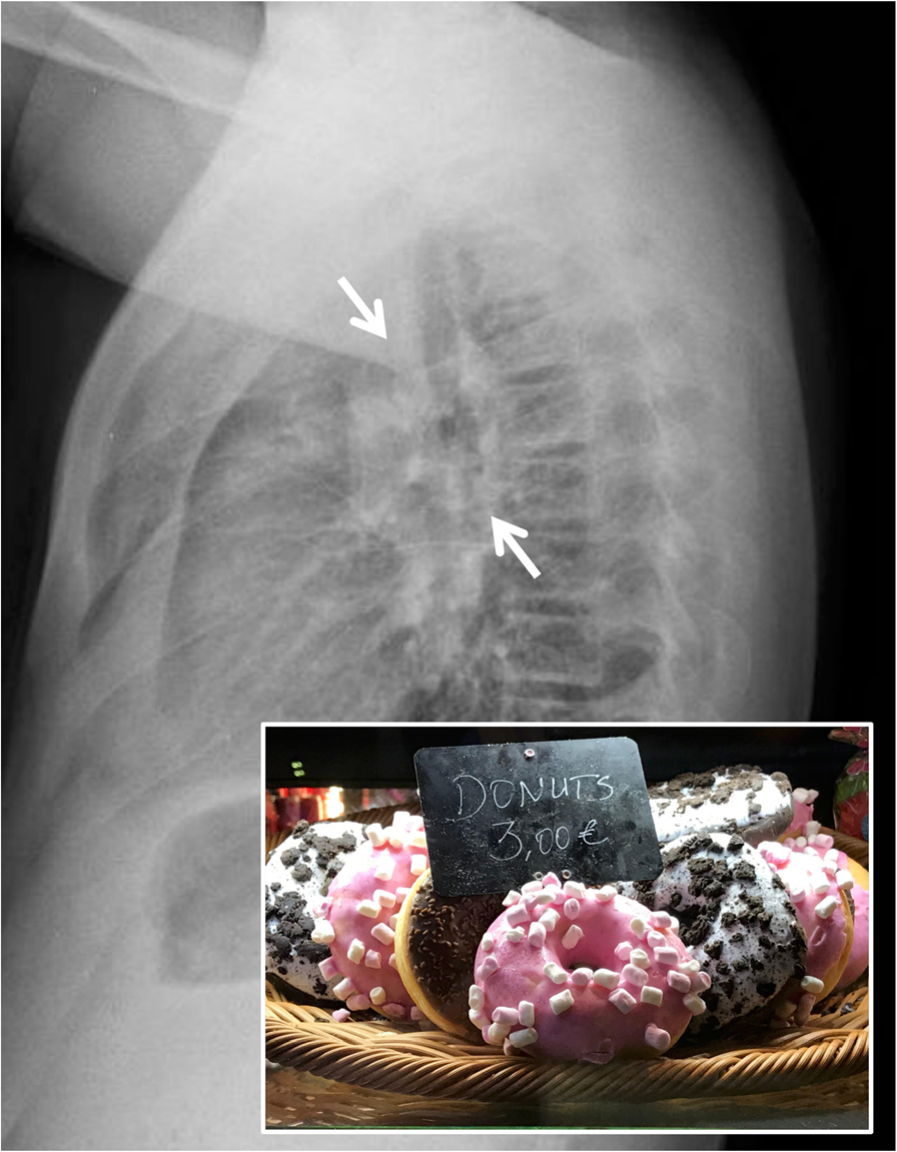
Doughnut sign. A lateral chest radiograph with the “doughnut sign”: the white arrows clearly demonstrate the presence of a white radiopaque ring at hilar region. It is created by enlarged lymph nodes at hilar and subcarinal region, located around intermedius bronchus, and normal profiles of right and left pulmonary arteries and aortic arch anteriorly and superiorly. This radiopaque ring resembles the appearance of a doughnut—as appreciable in the embedded figure. This sign could be suggestive of lymphadenopathy—in case of tuberculosis

The “doughnut sign” is recognizable in the latero-lateral projection of a chest radiograph or in the lateral projection of the CT scout: it consists of a complete radiopaque ring, which resembles a doughnut (Fig. 9) [6]. It is reproduced by normal profiles of right and left pulmonary arteries and aortic arch anteriorly and superiorly and by lymphadenomegaly inferiorly. The lymph nodes that complete the radiopaque ring are those of the subcarinal, hilar, and retrocarinal mediastinal sites. The radiolucent center of the “doughnut” consists of the trachea and the bronchi for the upper lobes. This sign is frequently found in cases of tuberculosis and lymphoma [6, 30].

### Eggshell calcifications

**Fig. 10:**
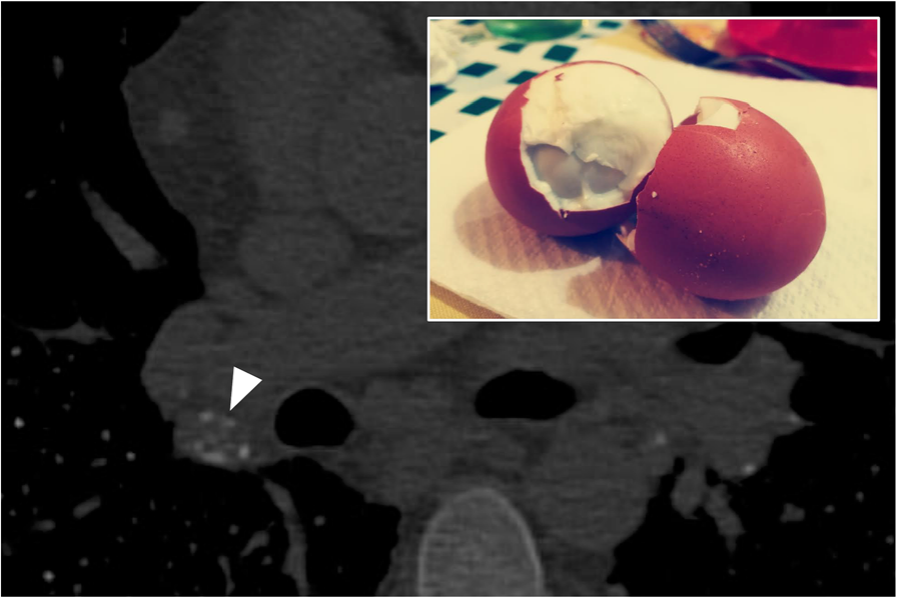
Eggshell calcifications. A small peripheral lamellar calcification (white arrowhead) of an enlarged lymphatic node, in a patient with silicosis; however, these calcifications, which reproduce the appearance of eggshells, are a non-specific sign of silicosis, since they may be encountered in various diseases, such as advanced sarcoidosis, pneumoconiosis, scleroderma, amyloidosis, lymphoma after radiotherapy, blastomycosis, and histoplasmosis

The “eggshell” calcifications can be observed on chest radiographs and CT images: they represent lymph nodes with lamellar calcifications (Fig. 10) and may be associated with different pathologies. The first descriptions in literature date back to the late ‘60s, by Jacobson and Felson [31]. According to the criteria published by Jacobson in 1967, the “eggshell” calcifications are present in the case of:

1. Calcifications arranged in the peripheral site, with a thickness of at least 2 mm, evident in at least 2 lymph nodes;
2. Calcifications that can be intact or “broken”;
3. At least one lymph node with a diameter greater than 1 cm;
4. At least one lymph node with a complete calcific ring;
5. Lymph nodes with peripheral calcifications and occasionally in the central part [31].

Eggshell calcifications are a non-specific sign which may be found in various diseases such as advanced sarcoidosis, silicosis, pneumoconiosis, scleroderma, amyloidosis, lymphoma after radiotherapy, blastomycosis, and histoplasmosis [31, 32].

### Feeding vessel sign

**Fig. 11:**
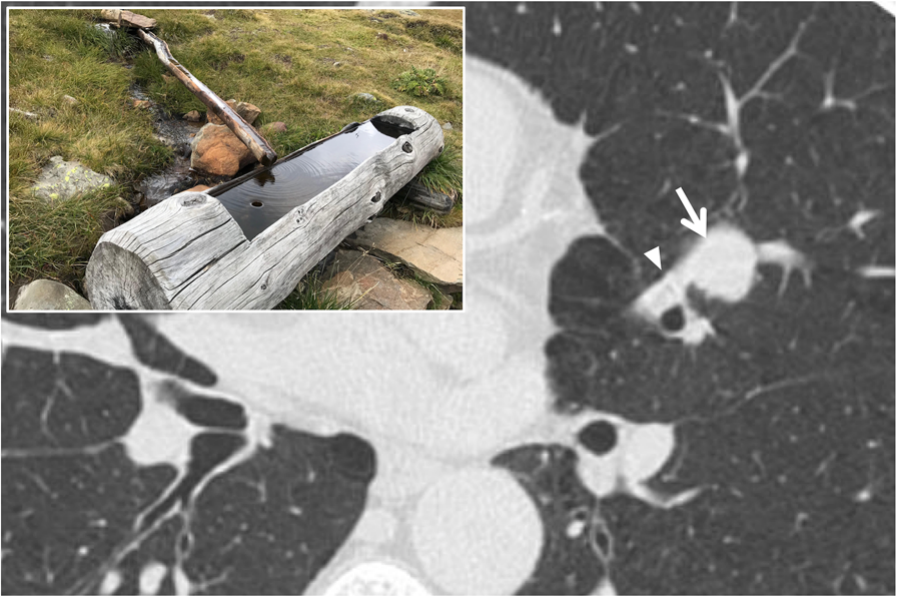
Feeding vessel sign. A patient with a pulmonary arterial-venous malformation (white arrow)—located in the upper left lobe; the respective feeding vessel (white arrowhead) is clearly depicted. More in detail, the “feeding vessel sign” could be described in cases of a pulmonary vascular branch that runs towards a focal lesion—“getting lost” in its context: it resembles the channel of water which provides adequate filling (as reproduced in the embedded figure)

The “feeding vessel sign” is produced by the presence of a pulmonary vascular branch that runs towards a focal lesion—“getting lost” in its context (Fig. 11). This radiological sign has two main meanings: (1) vascular origin of the lesion (for example, in cases of arterio-venous malformations or embolism) and (2) neoplastic nature of the lesion, with high neoangiogenetic activity. The sign, also known as “fruits on the branch sign”, has been associated with the presence of metastases or septic emboli [33, 34]; it is also frequently observed in pulmonary infarcts or arterio-venous fistulas [33]. In 1992, Murata et al. performed a pathological correlation study on this sign: the authors showed that in case of neoplastic nodular lesions, and especially in metastatic ones, the “feeding vessel sign” is determined by an arterial vessel that penetrates lesion only in 18% of cases in which this sign is detectable on CT examination; in 58% of cases, the vessel was dislocated by the lesion, proceeding immediately peripheral to the nodule [35]. As reported by Yudin in 2014, the feeding vessel sign is rarely detected in lung tumors or in granulomas [33].

### Finger-in-glove sign

**Fig. 12:**
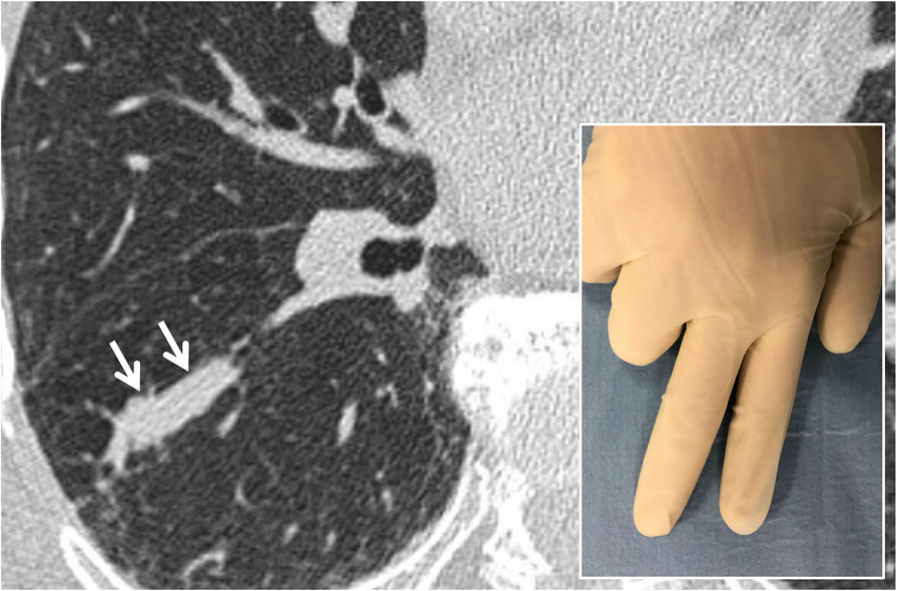
Finger-in-glove sign. Axial CT scan of a patient with bronchocele—located in the right lower lobe (white arrows). A bronchocele consists of a pulmonary opacity with characteristic shape (linear, V or Y shape)—with well-defined lobulated margins: the appearance is similar to that of a finger-in-glove

The finger-in-glove appearance is a sign that may be seen both on chest radiographs and in CT acquisitions; it consists of pulmonary opacities with characteristic shape (linear, V or Y shape) and well-defined lobulated margins (Fig. 12). This sign is due to dilation of bronchial structures filled by mucoid material (bronchocele). The first definition in literature dates back to 2003, when Nguyen described in the posterior-anterior projection of a chest radiograph linear or branched opacities extended from the hilum towards the periphery of the lung [36]. The “finger-in-glove” sign can be found mainly in two conditions:

1. Inflammatory diseases—such as cystic fibrosis, allergic bronchopulmonary aspergillosis, and asthma;
2. Obstructive diseases, both congenital (bronchial malformations) and acquired (foreign body) [36].

### Fleischner sign

**Fig. 13:**
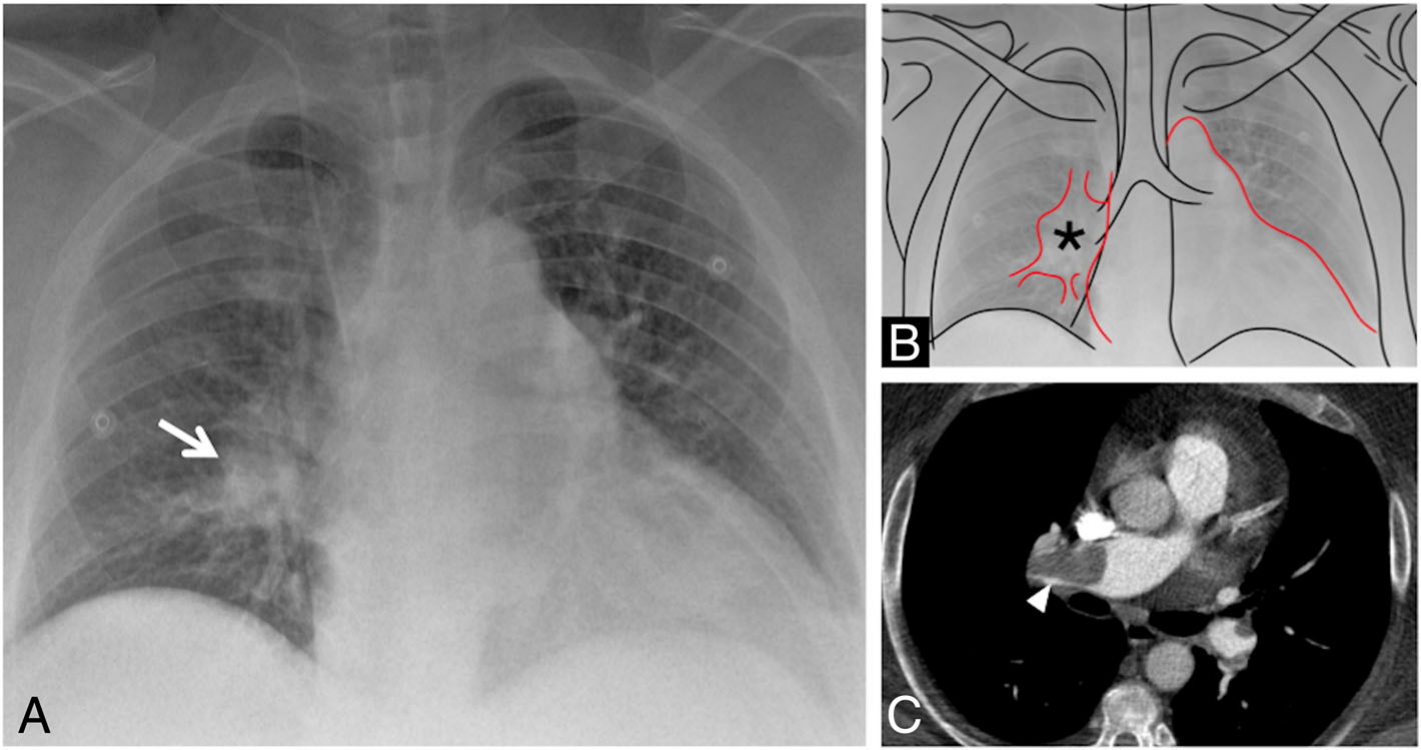
Fleischner sign. The “Fleischner sign” consists of a prominent central pulmonary artery upstream of cutoff (white arrows in **a**, black asterisk in **b**). It is frequently caused by a large embolus into the central pulmonary artery, as well depicted on axial CT image of the same patient (**c**, white arrowhead)

The “Fleischner sign” consists of a prominent central pulmonary artery upstream of cutoff (Fig. 13) [37]. It is caused by a large embolus into the central pulmonary artery, or it could be observed in case of pulmonary hypertension [38]. It was described by Felix George Fleischner in 1961 [39] and represents one of the most important radiological signs of pulmonary embolism seen on chest radiograph.

**Fig. 14:**
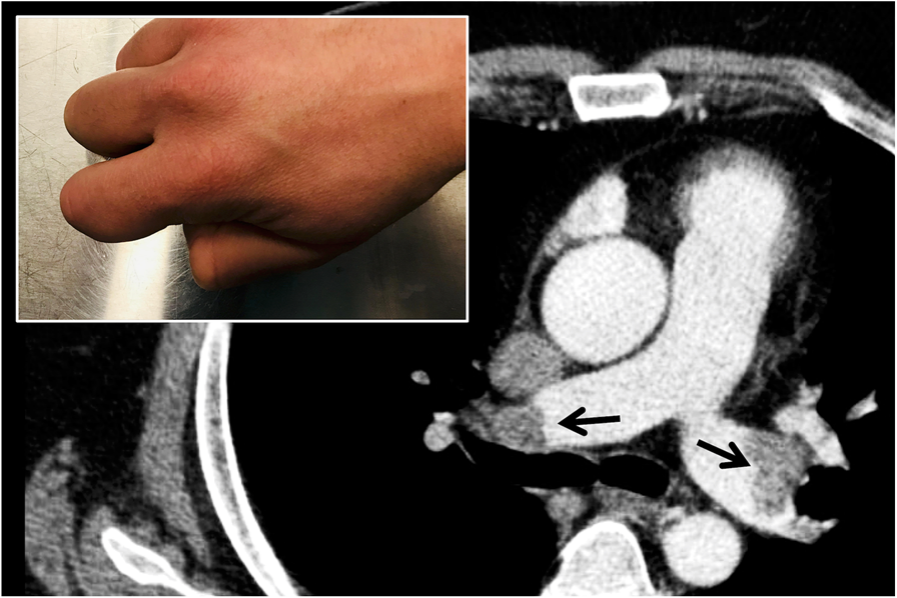
Knuckle sign. The figure clearly shows evident blood clot (black arrows) due to pulmonary embolism causing abrupt interruption of pulmonary arteries—in a patient affected by deep vein thrombosis. The abrupt interruption evokes the appearance of a knuckle

It is often associated with the “knuckle sign,” which is referred to an abrupt interruption of pulmonary artery’s branch, due to the presence of a blood clot—in a way that resembles the knuckle of a fist (Fig. 14). This sign is better seen on CT images; it has been described as one of the most typical signs of pulmonary embolism [37, 40].

### Galaxy sign

**Fig. 15:**
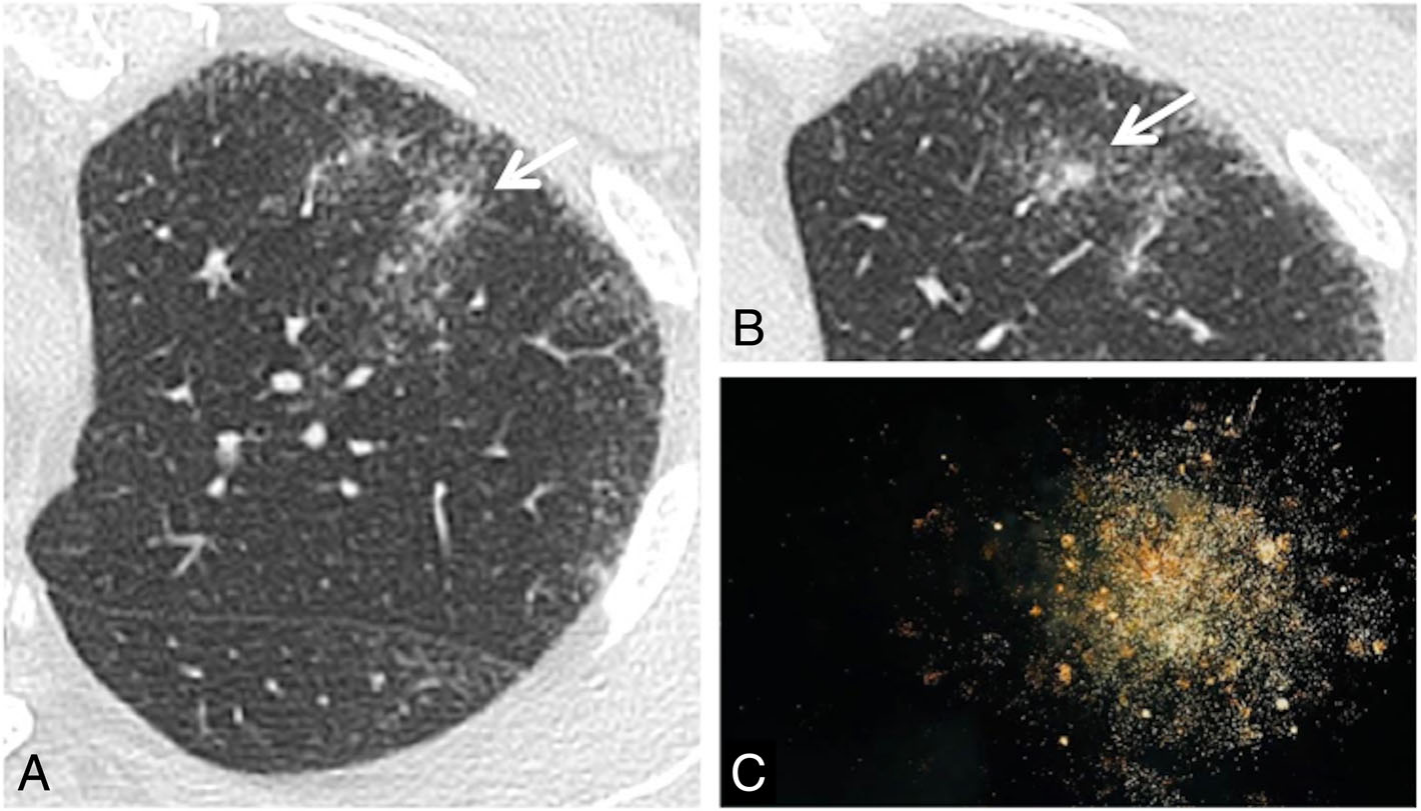
Galaxy sign. The figure shows two ill-defined nodular consolidations in the left upper lobe, surrounded by satellite small nodules (white arrows in **a** and **b**). The presence of small nodules—close to the central nodular areas—resemble the appearance of a “galaxy” (evocated in the embedded figure **c**). This galaxy appearance is a typical finding of sarcoidosis

The “galaxy sign” is a typical finding of sarcoidosis seen on MDCT acquisitions; it consists of a central lesion, typically > 1 cm, surrounded by satellite small nodules (Fig. 15); in some cases, it is also possible to depict ground glass attenuation areas. The galaxy sign has been defined and mentioned for the first time in literature by Nakatsu et al.—who described it in 2002; these authors also showed at CT-pathological correlation—explaining that such “sarcoid masses” are determined by multiple confluent small granulomas, which are better distinguishable at the periphery of the lesions. Nakatsu et al. described it in their series of cases of cavitation of these masses, a finding considered to be rather rare in these patients [41].

The galaxy sign may be also related to infectious diseases: large nodules arising from the confluence of small nodules have been also described in cases of active tuberculosis, namely when associated to tree-in-bud lesions [42].

### Ginkgo leaf sign

**Fig. 16:**
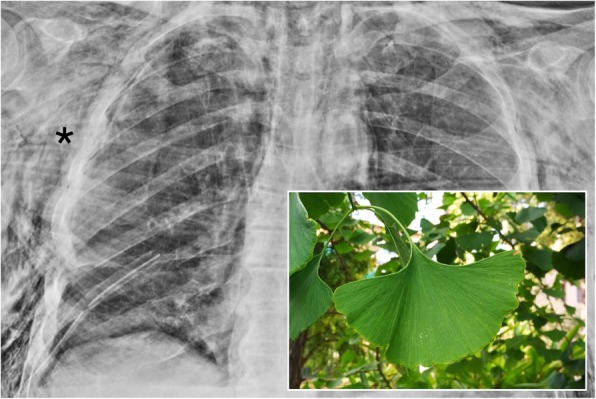
Ginkgo leaf sign. A chest radiograph of a patient with diffuse subcutaneous emphysema. The post-surgical radiograph clearly shows a large amount of air located in the subcutaneous tissues, penetrating into the pectoral muscle fibers (black asterisk). This striated appearance resembles a ginkgo leaf

The “ginkgo leaf sign” is detectable in the anterior-posterior projection of a chest radiograph: it is caused by the presence of large amount of air inside subcutaneous tissues of the chest wall (Fig. 16). Gas outlines the fibers of the pectoralis major muscle, creating a pattern that resembles the branching in the veins of a ginkgo leaf [43, 44]. In literature, it has been described as a typical imaging finding of patients with thoracic polytrauma [43].

### Golden S-sign

**Fig. 17:**
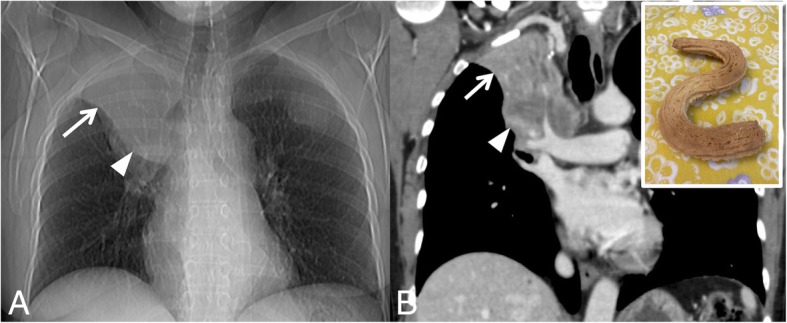
Golden S sign. The Golden S-sign reproduced in a patient affected by right pulmonary carcinoma. An “S” profile is clearly shown in **a**: this appearance is created by the right upper lobe atelectasis (superior and lateral part of the S profile) and by pulmonary neoplasm at the right hilum (inferior and medial part of the S profile). The same appearance is well depicted in coronal MPR CT image (**b**), very similar to the “S” profile of the biscuit reproduced in the embedded figure

The golden S-sign consists of an “S” profile reproduced on posterior-anterior chest radiograph by the presence of right upper lobe atelectasis with mass at right hilum. It has been also called “reverse S sign of Golden” and has been first described by Ross and Golden in 1925, who highlighted a right upper lobe collapse due to the presence of bronchogenic carcinoma of the right hilum [45]. On chest radiographs, the superior and lateral part (concave inferiorly) of the “S” profile is represented by the upper lobe collapse, whereas the inferior and medial part (convex inferiorly) may be explained by the associated pulmonary mass [46, 47]. This sign may be more easily appreciated on MDCT (Fig. 17), as recently reported in literature [46, 48]; it can be also recognizable not only in cases of pulmonary bronchogenic carcinoma, but also in cases of lymphadenopathy or mediastinal tumors.

### Halo sign

**Fig. 18:**
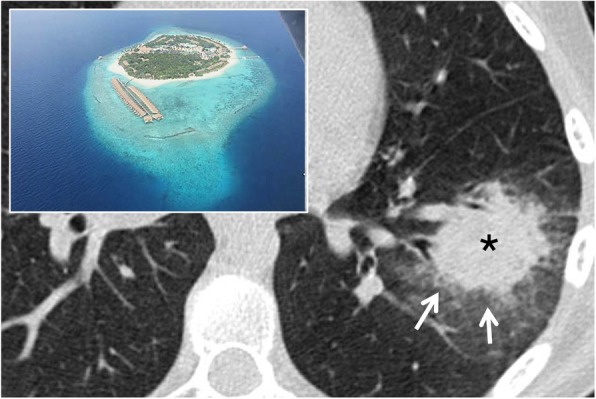
Halo sign. The CT image shows the “halo sign” in a patient with angioinvasive aspergillosis: in the left lower lobe, it is possible to appreciate a round consolidation (black asterisk) with peripheral ground glass (white arrows), which suggests a hemorrhagic process. This ring or peripheral ground glass is similar to the sea’s appearance adjacent to an atoll

The “halo sign” may be highlighted in MDCT when a solid lesion is surrounded by a peripheral ground glass area (Fig. 18). The ground glass attenuation, in most cases, is considered to be a perilesional hemorrhagic process.

Kuhlman et al. described this sign in 1988 as characteristic of angio-invasive aspergillosis [49], although over time this finding was associated with a broader spectrum of possible pathologies, such as neoplastic lesions and other non-neoplastic and non-infectious conditions (vasculitis, organizing pneumonia, pulmonary endometriosis) [50].

Although the halo sign has been reported thicker for infectious diseases [51], the clinical context may be very helpful to achieve a differential diagnosis among diseases that reproduce this CT sign. In immunocompromised patients, the halo sign has been associated with fungal infection (invasive aspergillosis, pulmonary candidiasis, coccidioidomycosis) and lymphoproliferative disorders; in immunocompetent patients, lesions that reproduce halo sign include primary neoplasms, metastases, vasculitis (Wegener), sarcoidosis, and organizing pneumonias [51, 52].

### Headcheese sign

**Fig. 19:**
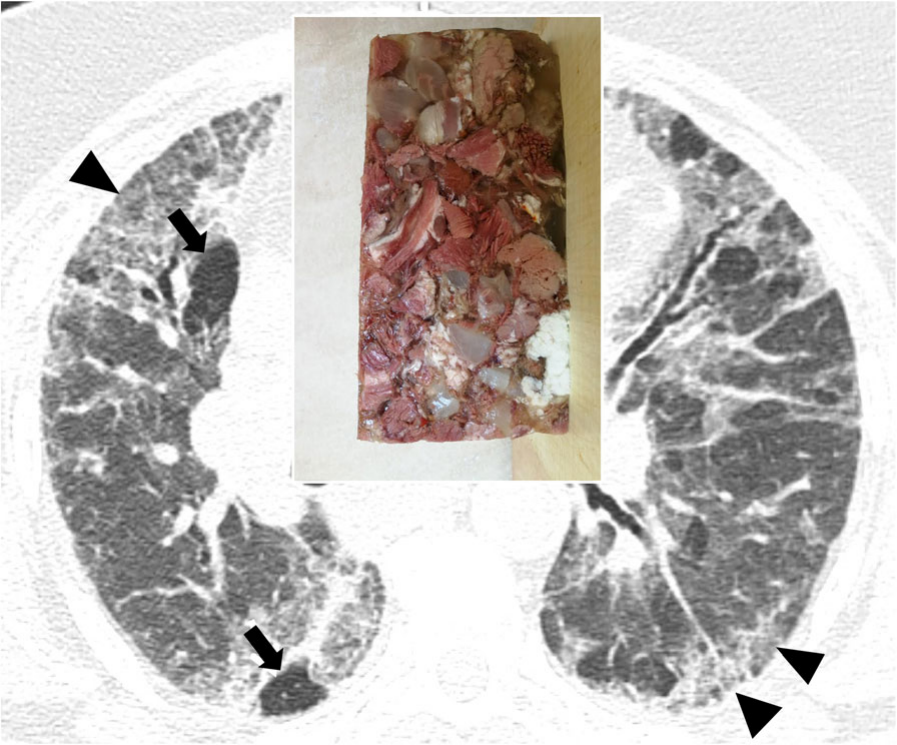
Headcheese sign. This sign consists in a mixed pulmonary pattern with areas of various attenuation. It is characterized by the contemporary presence of areas of ground glass attenuation (black peripheral arrowheads, due to infiltrative diseases), air-trapping (black arrows), and healthy lung zones. This pattern is reminiscent of the appearance of headcheese—as well demonstrated in this patient with subacute hypersensitivity pneumonitis

The so called “headcheese” sign was introduced to the literature by Patel Rita et al., who had described this imaging appearance in 2000 as a typical feature of hypersensitivity pneumonitis [53]. This imaging pattern may be produced—on CT images—by the simultaneous presence of ground glass opacities, air-trapping areas, and healthy lung regions (Fig. 19); normally, this sign may be seen on expiratory CT images. It can be seen when obstructive small-airway disease coexists with an alveolar inflammatory infiltration component [54]. It has been considered pathognomonic of CHP (chronic hypersensitivity pneumonitis), even if this sign may actually occur in other pathological conditions—such as infections associated with bronchiolitis or in some atypical cases of sarcoidosis with alveolar involvement. The headcheese sign definition derives from the CT appearance of the pulmonary parenchyma, where is possible to appreciate the presence of lobular areas with different densities—resembling that of the typical European meat dish [54].

### Honeycombing

**Fig. 20:**
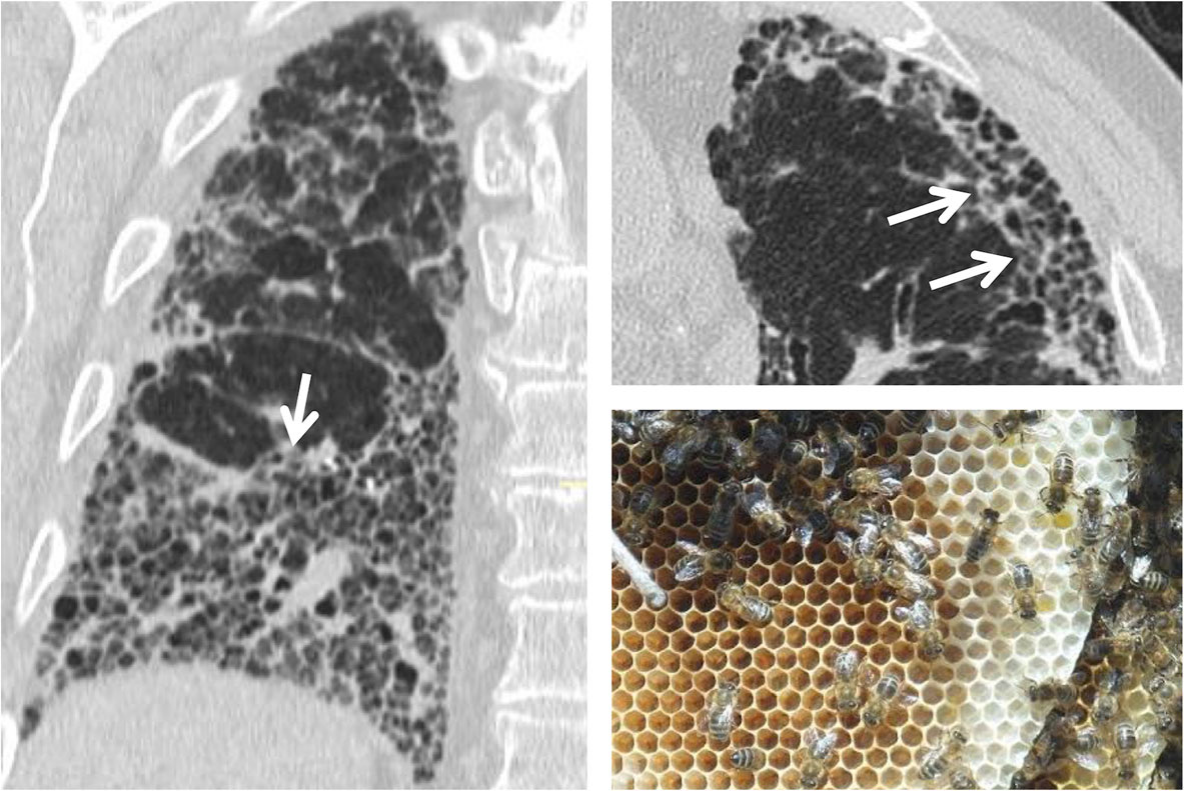
Honeycombing. The figure shows a patient affected by UIP: this radiological pattern is characterized by traction bronchiectasis, honeycombing, and reticulations—frequently recognizable in basal and subpleural regions. The small cystic areas—seen on coronal scan (left image) and axial scan (right image) is very similar to a honeycomb

The term “honeycomb appearance of the lung” seems to originate in Germany in the mid-nineteenth century: however, at that time, it was mainly used to describe the appearance of bronchial malformations or conglomerations of cystic bronchiectasis. Later, other authors described the characteristic appearance of UIP (usual interstitial pneumonia) as “bronchial emphysema” or “cystic pulmonary cirrhosis”. The first use of the term “honeycomb lung”—in the meaning accepted today—is attributed to Oswald and Parkinson, who used it and defined its characteristics in 1949 [55]. According to the Fleischner Society glossary, honeycombing or honeycomb-like appearance is characterized by the presence of cystic spaces with a wall, located in subpleural regions, showing a diameter between 3 and 10 mm (Fig. 20) [1]. It represents the final stage of parenchymal fibrotic changes and a total subversion of the lung architecture. Honeycombing tends to affect more frequently the basal and peripheral regions of the lung parenchyma, and when extended, it is considered a highly suggestive sign of UIP. However, it should be remembered that some areas of honeycombing can be found in other fibrous pathologies, such as chronic hypersensitivity pneumonia, fibrotic stage of sarcoidosis (stage IV), and pneumonias; however, in these cases, the areas of cystic degeneration are significantly less extensive and characteristically show atypical localizations (e.g., greater involvement of the mid-apical parenchyma). Although to define the presence of honeycombing, the cystic spaces had to be organized on several strands; the recent White Paper of the Fleischner Society has partially modified this feature, stating that even a few subpleural cysts with a wall on a single layer, in the correct pathological context, can be defined as areas of honeycombing [56].

### Interface sign

**Fig. 21:**
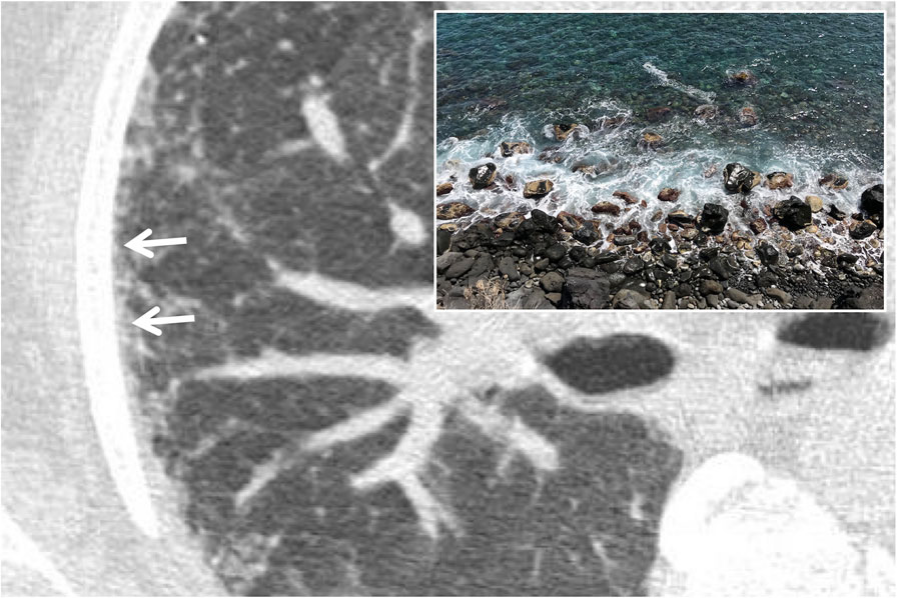
Interface sign. Irregular appearance of pleural surface due to fibrosis (white arrows) in a case of UIP. In cases of interstitial disease with fibrous component, interstitial thickening with focal retractions of the pulmonary parenchyma may develop—creating an alteration of the regularity of the profiles between lung parenchyma, vessels, bronchi, or visceral pleura. In these cases, it is possible to observe a “jagged appearance”

The interface sign represents a CT finding encountered in fibrosing interstitial lung diseases [57]. Normally, the interface between subpleural/mediastinal fat and pulmonary parenchyma appears linear and regular; when an interstitial disease with a fibrous component is present, interstitial thickening with focal retractions of the pulmonary parenchyma may develop—creating an alteration of the regularity of the profiles between lung parenchyma, vessels, bronchi or visceral pleura. In these cases, it is possible to observe a “jagged appearance” (Fig. 21). The interface sign may be found in all fibrosing lung diseases—such as UIP, NSIP, and CHP—being one of the most significant signs in recognizing these pathological conditions [58].

### Monod sign

**Fig. 22:**
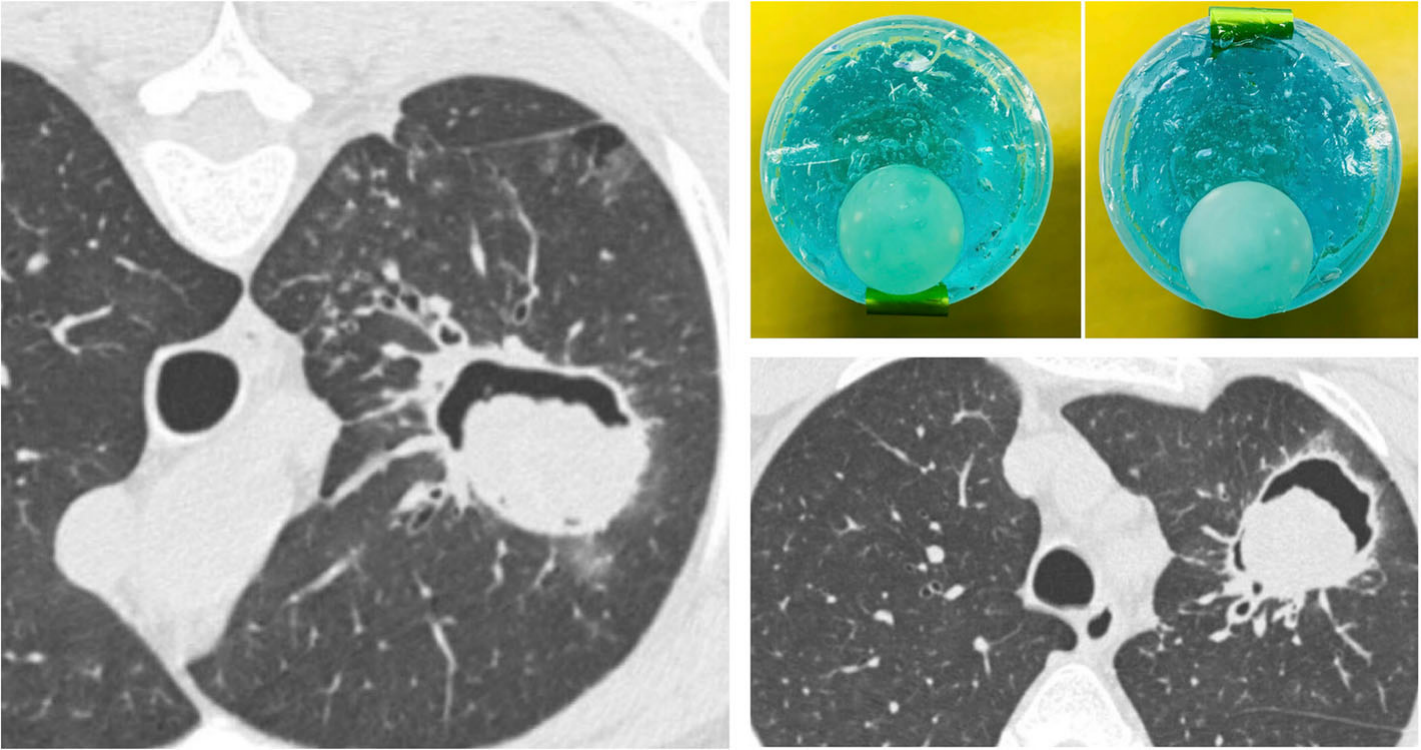
Monod sign. Aspergilloma (asterisk) into a cavitated lesion located in a left upper lobe. The fungus mass moves according to the prone (left) and or supine (bottom left) patient position

The “monod sign”—firstly described by Pesle and Monod in 1954—refers to air surrounding a mycetoma (usually an aspergilloma) in a pre-existing cavity (Fig. 22) [59]. It has a different prognostic meaning than the air crescent sign [6].

The monod sign of aspergilloma is found in immunocompetent patients, with a history of previous cavitating or cystic lung diseases; in most cases, these are patients with previous tuberculosis. The symptomatology is usually characterized by hemoptysis. In case of aspergilloma, the mass (consisting of fungal hyphae, mucus, and cellular debris) inside the cavity appears to be mobile, following the changes in the patient position: this allows us to distinguish a mass inside a cavity (monod sign) from a cavitated mass [60, 61].

### Mosaic attenuation

**Fig. 23:**
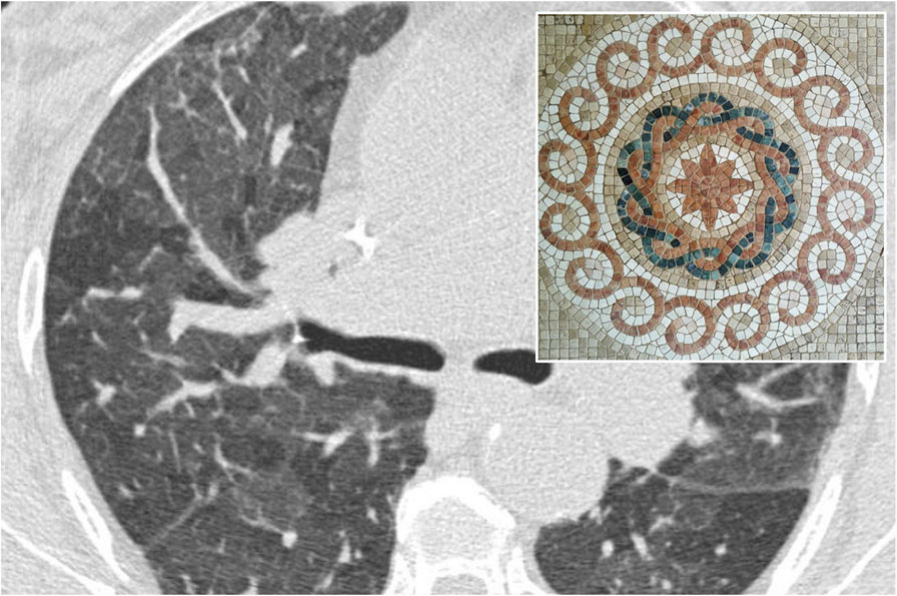
Mosaic attenuation. Mosaic pattern in a patient affected by asthma. Lung parenchyma shows different attenuation areas, due to presence of pulmonary lobules with decreased density, caused by air-trapping. This appearance recalls a mosaic, as clearly evident in the embedded figure.

On CT images, the “mosaic attenuation” pattern refers to an aspect of the lung in which areas with different attenuation alternate each other (Fig. 23). It was first described in 1993 by Eber et al. as a suggestive pattern of bronchiolitis [62] and in 1994 by King et al. as a typical aspect of pulmonary parenchyma in cases of chronic pulmonary embolism [63]. It must be borne in mind that a minimum degree of inhomogeneity of parenchymal attenuation can be found in physiological conditions: the sloping portions of the lung, for example, show a greater attenuation, as well as greater perfusion (and therefore, greater attenuation) may be observed in the central portions rather than the peripheral ones. The mosaic pattern is a nonspecific sign, which may be found in various pathological conditions: obstruction of the small airways, occlusive vascular pathology, and parenchymal pathology. It is possible to differentiate the various pathological conditions through some CT features. When in the context of hypo-attenuation areas there are smaller vessels than other pulmonary regions, the cause of mosaic appearance could be vascular (for example, chronic pulmonary embolism); in this condition, the hypodense areas represent the areas of abnormality. When vessels are uniformly represented in pulmonary regions with different attenuation, expiratory scans may be helpful for radiologists. In expiration, if hypo-attenuation areas do not show an increase in density, the lung contains “air trapping” areas—due to a pathology of the small airways (bronchiolitis, for example); also in this condition, the hypodense areas correspond to the areas of abnormality. In case of primarily parenchymal disease, an infiltrative process occurs in the pulmonary interstitium, with the alveoli filling with fluid, cells, or fibrosis: in this condition, the portions of the affected parenchyma show greater attenuation than areas of healthy lung. The caliber and the number of vessels do not differ between the pathological and healthy areas. This kind of mosaic pattern may be found in *Pneumocystis jirovecii*’s pneumonia [64, 65].

### Oreo cookie sign

**Fig. 24:**
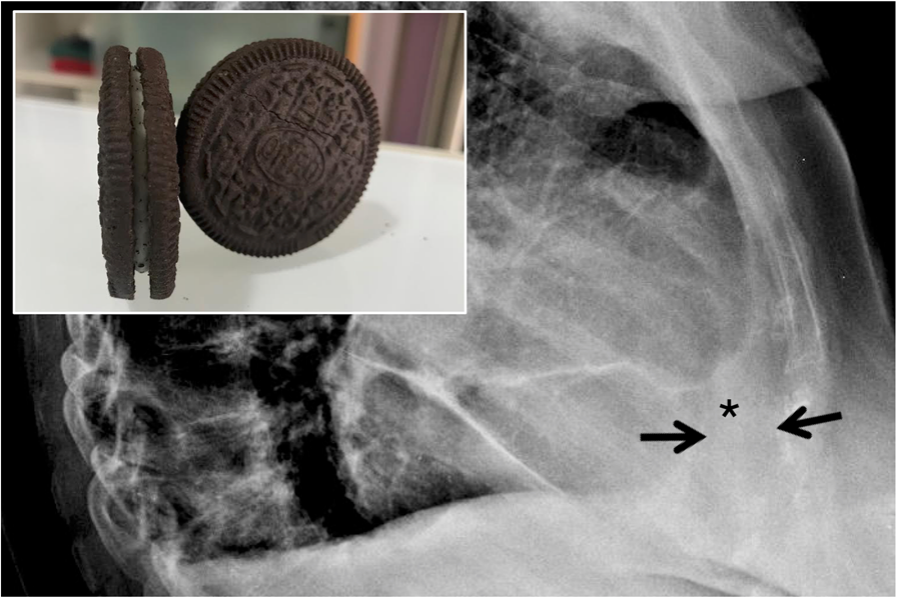
Oreo cookie sign. On lateral chest radiograph the Oreo cookie sign is depicted: it consists of an increased radiopacity of the pericardium (black asterisk)—caused by pericardial effusion—which appears to be anteriorly and posteriorly delimited by two radiolucent lines (black arrows), caused by pericardial and epicardial fat. This radiographic pattern reproduces the so called “Oreo cookie sign,” similar to the famous biscuit

Described in the title of a recent paper—the “Oreo cookie sign” refers to the aspect of the pericardial effusion which may be seen on the lateral chest radiograph (Fig. 24). Typically, the pericardial effusion causes an increased radiopacity of the pericardium, which appears to be anteriorly and posteriorly delimited by two radiolucent lines. These lines correspond respectively to the pericardial fat and the epicardial fat [66]. It may be better and more often seen on CT images.

### Polo mint sign

**Fig. 25:**
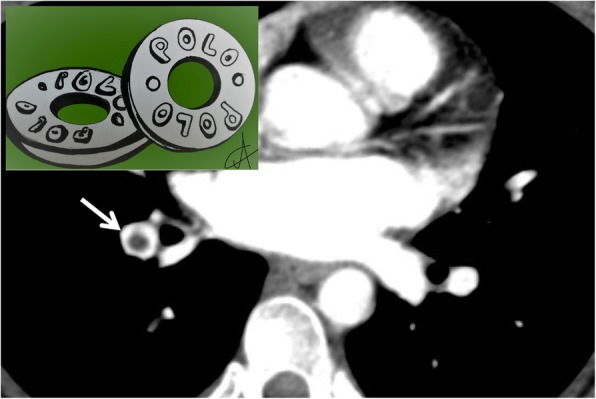
Polo mint sign. A patient with acute pulmonary embolism: a blood clot (white arrow) surrounded by contrast media, reproduces inside the pulmonary vessel the “polo mint sign” appearance (similar to the famous polo mint in the embedded figure). Its recognition is very important for radiologists, because it represents a marker of acute embolism

In the thoracic area, the “polo mint sign” refers to the typical aspect of acute pulmonary embolism, when the thrombosed vessel is seen on axial planes (Fig. 25). It has been described in a paper published in 2004 by Wittram et al., who described imaging findings of acute pulmonary embolism [67]. The polo mint sign corresponds to the railway track sign, which instead describes the thrombosed vessel displayed according to a plane parallel to its major axis. It is found in contrast-enhanced CT examinations and is given by the presence of contrast material surrounding a central filling defect [67].

### Popcorn calcification

**Fig. 26:**
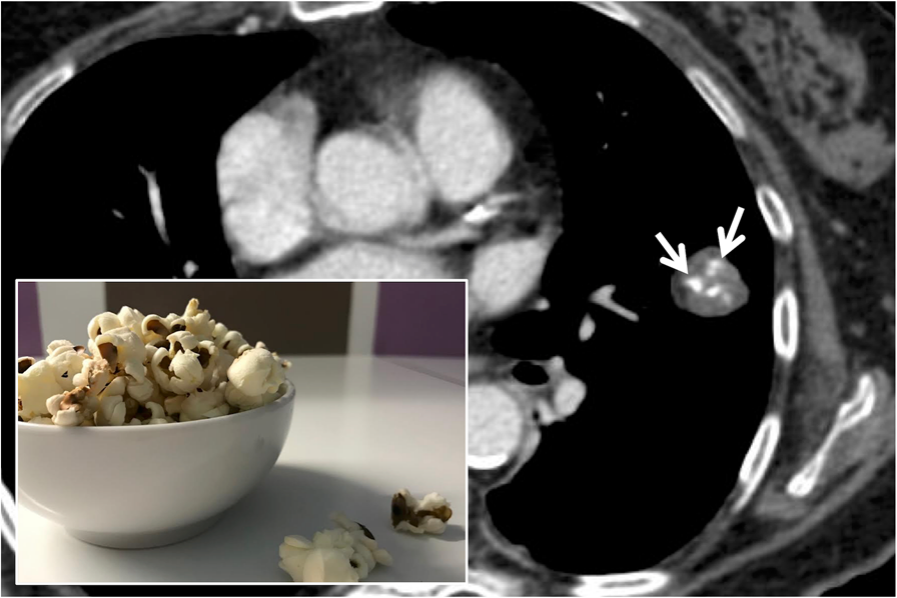
Popcorn calcification. Popcorn calcifications may be encountered in cases of patients with pulmonary hamartoma. The figure shows the presence of a pulmonary hamartoma, which is characterized by the presence of fat and amorphous calcification (white arrow), which remind us of the appearance of a piece of popcorn

This sign refers to the presence of amorphous calcifications, often ring-shaped, which remind us of the appearance of a piece of popcorn (Fig. 26). In the pulmonary domain, popcorn calcifications within a well-defined nodule suggest a diagnosis of benignity, namely hamartoma. According to the study by Briccoli et al.—published in 1993—popcorn calcifications would be present only in 10% of pulmonary hamartomas [68]. They can be detected on chest radiographs but better on pulmonary CT scan, which also allows for the identification of intra-lesional fat (in about 60% of cases). The adipose tissue usually appears organized in small groupings dispersed in the calcification field. Other types of suggestive calcifications of benignity are widespread, centralized, and stratified calcifications; punctate and eccentric calcifications are more often associated with malignant lesions (carcinoid or osteosarcoma metastasis) [69, 70].

### Positive bronchus sign

**Fig. 27:**
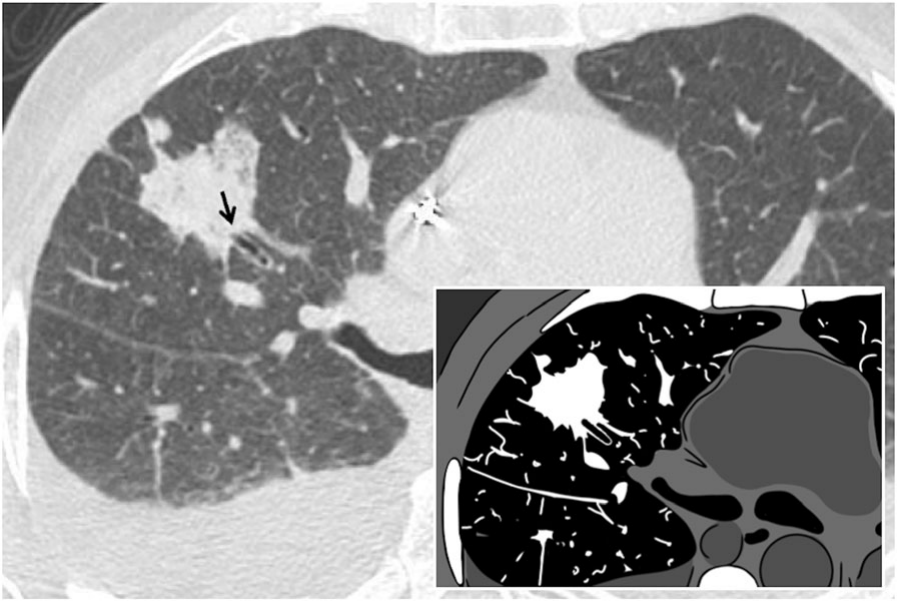
Positive bronchus sign. The chest CT images and the embedded drawing demonstrate the positive bronchus sign: it consists of an air-filled bronchus (black arrow)—seen as a tubular hypo-attenuation area—oriented towards a peripheral nodular formation

On chest CT images, the positive bronchus sign consists of an air-filled bronchus—seen as a tubular hypo-attenuation area—oriented towards a peripheral nodular formation (Fig. 27). In 1988, Naidich et al. highlighted the importance of the positive bronchus sign inside a peripheral nodule: prior to a possible bronchoscopy, this finding would be predictive of a diagnostic result of the examination itself [71].

A clear explanation of this radiological sign has been reported in literature by Singh, in 1998 [72]. The hypo-attenuation area may extend into the nodule—producing an air bronchogram. The bronchus sign is not found in all types of lesion, more frequently seen in masses (≥ 3 cm) and in those with spiculated margins. Several studies have shown that this sign is more often associated with malignant lesions and, in particular, with pulmonary adenocarcinoma with lepidic pattern and adenocarcinoma. The relationship between the affected bronchus and the mass, however, may present variants. Tsuboi et al. have described four types: (1) the bronchus reaches the nodule and undergoes an interruption; (2) the bronchus arrives at the nodule and continues inside it; (3) the bronchus is compressed by the tumor, maintaining however the intact mucosa; and (4) the bronchus is obstructed due to the submucosal spread of the tumor, causing an irregular thinning of the same. The description of the relationship between bronchus and lesion plays an important role in the diagnostic planning, since in the first two types biopsy is indicated; in the other two cases, better results are obtained using transbronchial aspiration [73, 74].

### Railway track sign

**Fig. 28:**
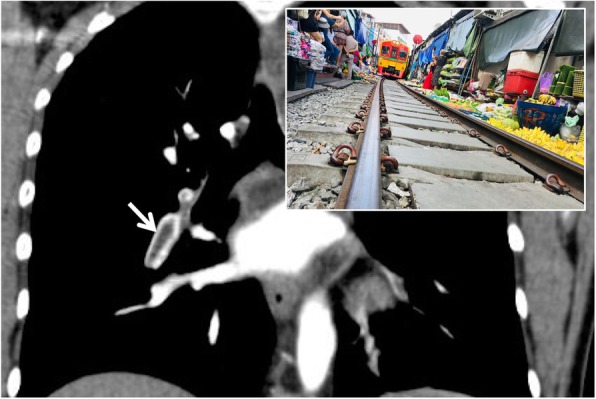
Railway track sign. The coronal CT image well depicts linear and centric filling defect (white arrow) inside the lumen of an arterial lung vessel—due to acute embolism; the contrast, surrounding the central endoluminal defect of opacification, reproduces a railway track appearance. The railway track sign is very similar to the polo mint sign, which instead describes the thrombosed vessel displayed according to an axial plane

The railway track sign is a radiological sign that may be found on CT images of patients with acute pulmonary embolism; it occurs when a pulmonary arterial vessel, seen in longitudinal section, presents a partial filling defect due to the presence of a thrombus placed centrally at the vessel and the contrast media disposed at the periphery, reproducing a typical “rail track” image (Fig. 28). This sign is strictly related to the “polo mint sign” (previously described) in which the thrombosed vessel is displayed in section perpendicular to the major axis. In cases of chronic pulmonary embolism, the “rail track” image is no longer visible because the thrombus is placed in an eccentric position to the vessel [63].

### Scimitar sign

**Fig. 29:**
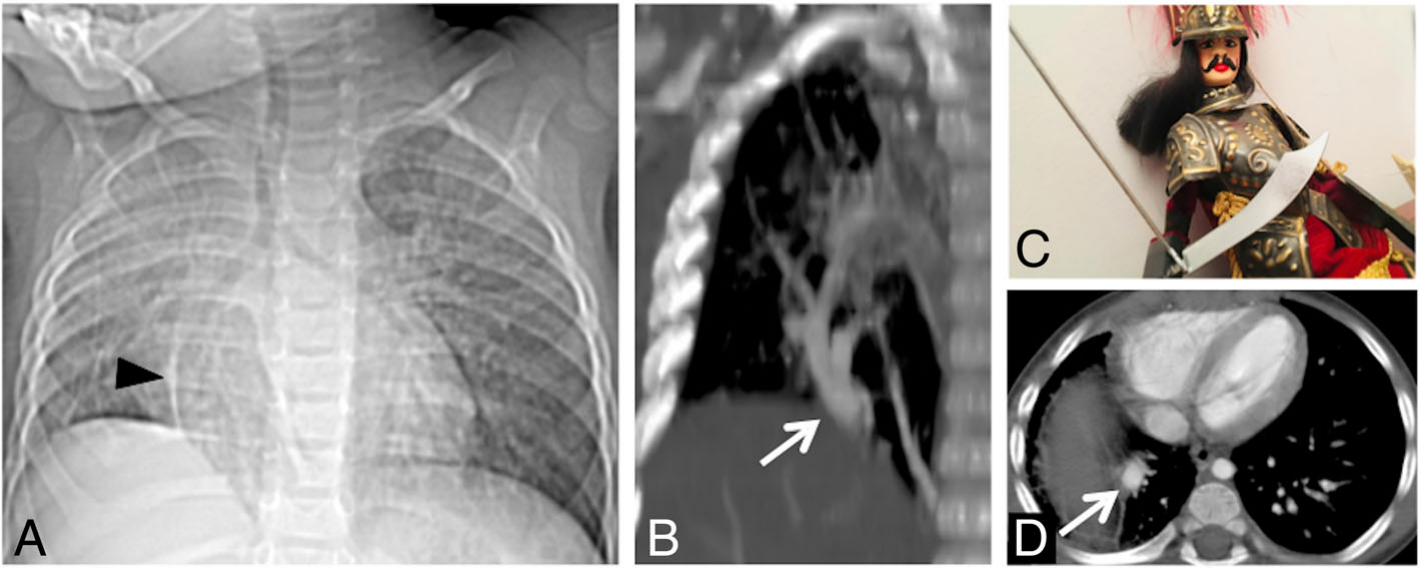
Scimitar sign. Scimitar syndrome in patients with abnormal pulmonary venous return. The scimitar sign (white arrows in **a** and **b**) can be easily recognized: the anomalous vessel often runs as a radiopaque tubular structure parallel to the right margin of the cardiac shadow (anterior-posterior CT scout and coronal CT reformatted images), reproducing the shape of the famous Turkish curved sword (**c**). On axial CT scan (**d**), the abnormal pulmonary vein is clearly recognizable (white arrow)

Scimitar syndrome is a rare cardiopulmonary anomaly, characterized by a left-right acyanotic shunt due to an abnormal pulmonary venous return. This condition—first described in 1962 [75]—is more frequently encountered in the right hemithorax and is associated with pulmonary hypoplasia. The abnormal vessel drains (a) in the inferior caval vein (more frequently), (b) in the right atrium, and (c) in a branch of the portal venous system. In about a third of cases, the scimitar sign (Fig. 29) can be recognized on chest radiographs of patients affected by this syndrome: the anomalous vessel often runs as a radiopaque tubular structure parallel to the right margin of the cardiac shadow, reproducing the shape of the famous Turkish curved sword. Other radiological features include the presence of a hypoplasic lung and of an ipsilateral mediastinal shift. CT angiography and MR angiography highlight this vascular abnormality very well and are very helpful in excluding pulmonary sequestration and pulmonary vein varices, which represent the main differential diagnosis [76].

### Signet ring sign

**Fig. 30:**
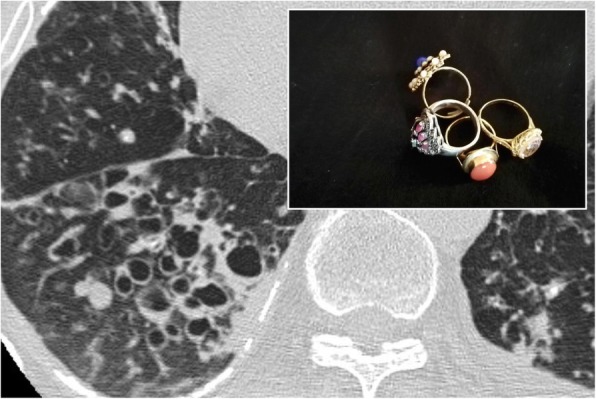
Signet ring sign. In a young patient affected by cystic fibrosis, multiple air-filled bronchiectasis are flanked by respective pulmonary vessels: this appearance reproduces the so-called signet ring sign

The “signet ring sign” represents a thoracic sign which may be seen in thoracic CT scan: it has been described in literature by Hugue Ouellette in 1999 [77]. The CT images demonstrate an ectatic bronchus with thickened walls and flanked by the respective pulmonary artery, seen in cross section, resembling the appearance of a ring with a signet (Fig. 30). The bronchus and the artery should have similar size in normal pulmonary parenchyma; in case of bronchiectasis, this ratio is altered, with increased size of the bronchus [77].

### Snowstorm sign

**Fig. 31:**
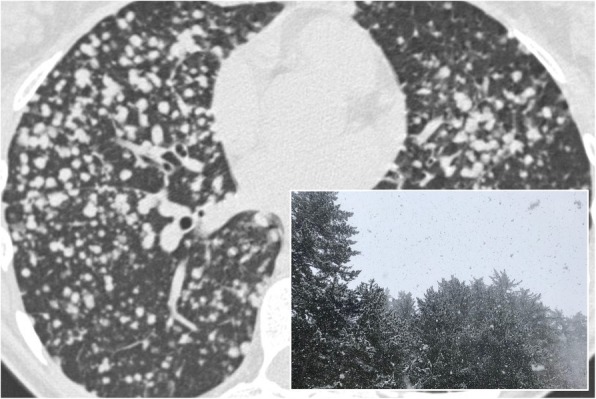
Snowstorm sign. Innumerable nodules diffuse through the lung parenchyma, in a patient with diffuse metastatic disease from renal cell carcinoma. The multiple nodules in the lung parenchyma suggest a snowstorm appearance, as reproduced in the embedded figure

The “snowstorm sign” or appearance is due to miliary diffusion of innumerable micronodules (1–2 mm) in all the pulmonary parenchyma (Fig. 31); this radiological feature may be appreciated on chest radiograph. Yudin et al. described this radiological appearance for small and innumerable metastatic nodules related to vascular tumors (thyroid gland carcinoma, renal cell carcinoma)—whereas they recommend the term cannonball metastases for lesions showing big size and accurately outlined, due to gastrointestinal tumors [78].

This radiological pattern has been also described in literature by Al-Thuhli et al., which reported a case of “snowstorm” appearance for a miliary distribution of metastatic nodules from papillary thyroid carcinoma [79]. More often, it requires MDCT scan for diagnostic confirmation, namely for nodules that are “truly miliary in size”: differential diagnosis include miliary tuberculosis, fungal infections (histoplasmosis, coccidioidomycosis), sarcoidosis, and chickenpox infection calcifications.

A pulmonary snowstorm has been also reported in literature by Bhalotra et al., for a case of a 28-year-old-man with diffuse lung micronodules due to pulmonary alveolar microlithiasis [80].

The study of nodule margins through MDCT scans helps in the differential diagnosis: poorly defined margins can be more easily associated with a distal involvement of the air spaces (acinar rosettes), whereas very defined ones suggest an interstitial pathology [78].

Clinical history, other radiological findings, or comorbidities may help radiologist in the diagnostic process: in some circumstances, the presence of these fine parenchymal micronodules is correlated to a neoplastic etiology in case of known primary tumor or to sarcoidosis in case of enlarged mediastinal lymphatic nodes.

### Sunburst sign

**Fig. 32:**
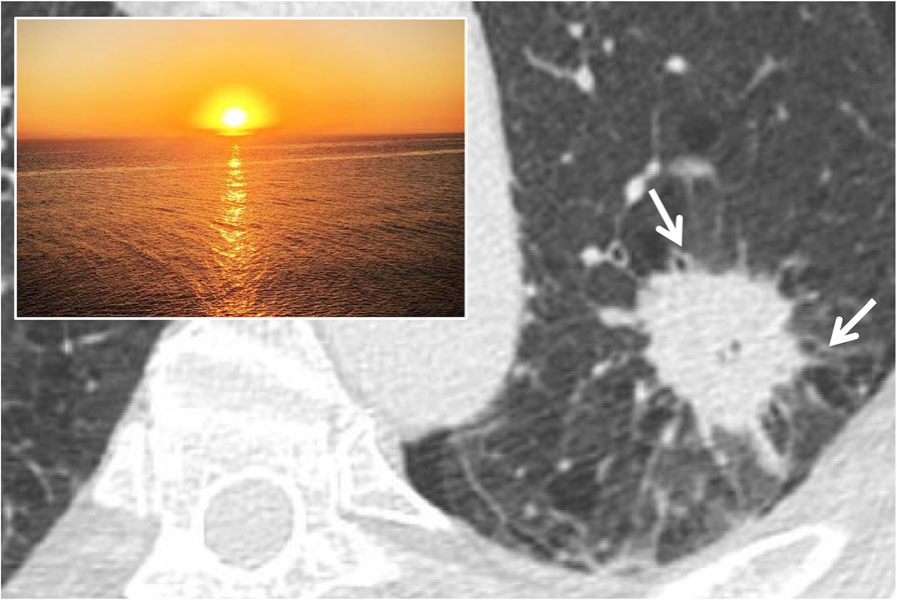
Sunburst sign. A CT image of a 61-year-old female affected by lung adenocarcinoma. The parenchymal neoplastic lesion shows spiculated and irregular margins, resembling the appearance of a “sunburst”: the rays or the speculated margins are constituted by the distorted blood vessels and/or by thickened septa that surround the pulmonary mass

The “sunburst sign” is represented by a pulmonary nodule or a parenchymal mass with irregular and spiculated margins, such as sunrays—hence the expression “sunburst sign”, reported in 1997 by O’donovan [81]. The rays or the spiculated margins are constituted by the distorted blood vessels and/or by thickened septa that surround the pulmonary nodule (Fig. 32). In the differential diagnosis, it is very important to include the galaxy sign, which refers to benign micronodules around a nodule of sarcoidosis. The sunburst sign is very suggestive of a malignant lesion, in particular for pulmonary adenocarcinoma; the presence of speculated margins is considered a risk factor for malignant neoplasia, with an odds ratio range of 2.2–2.5 concerning nodules obtained from screening studies [82].

### Tree-in-bud

**Fig. 33:**
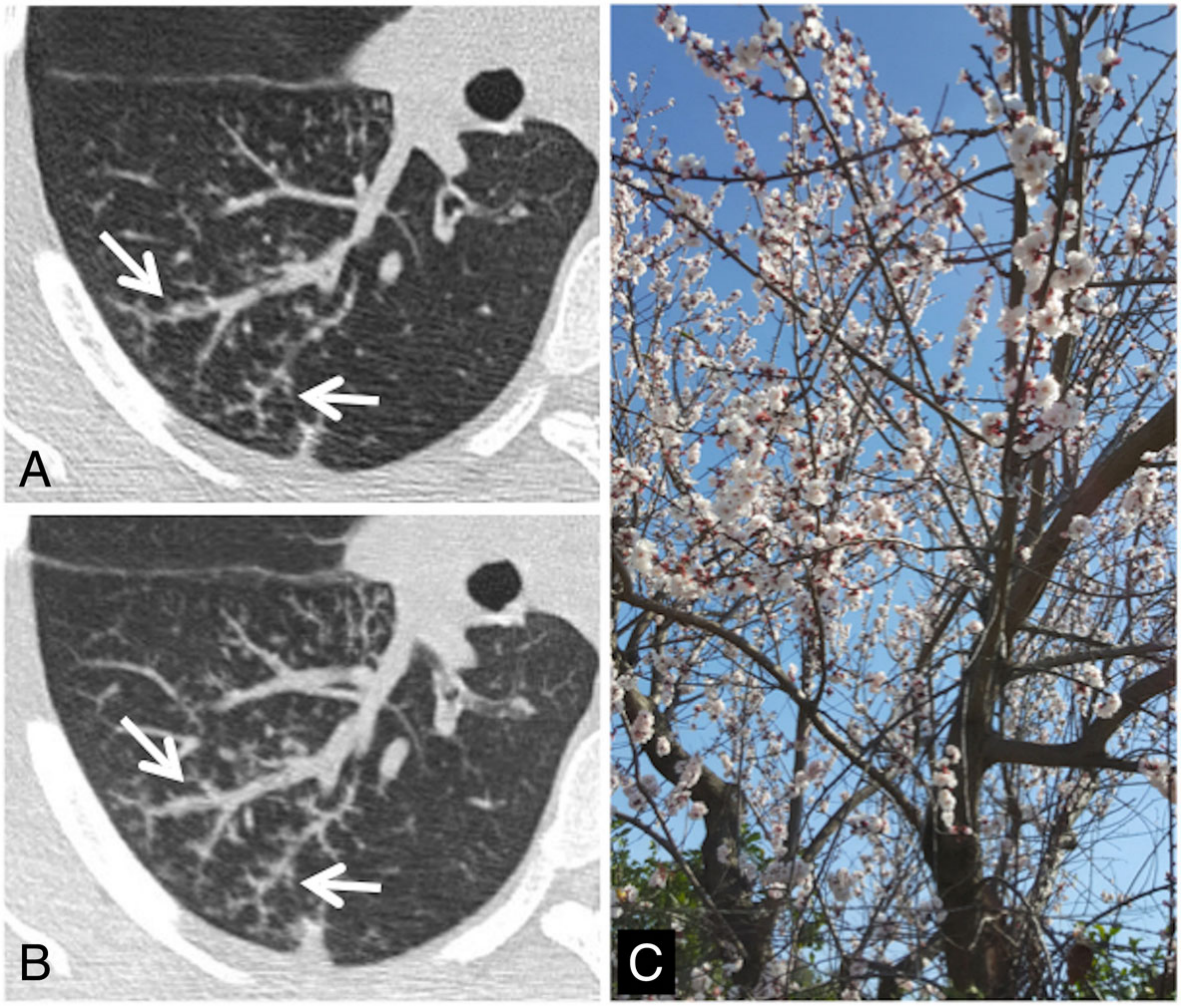
Tree-in bud sign. Small centrilobular nodularities and linear ramifications (white arrows in **a** and **b**) may be appreciated in the right lower lobe of a patient with cough and fever; these CT findings—which show the typical appearance of tree-in-bud—are related to the presence of inflammatory bronchiolitis. This tree-in-bud appearance is a morphological pattern that resembles a flowering tree (**c**)

On MDCT images, the tree-in-bud appearance is a morphological pattern that resembles a flowering tree (Fig. 33); it is characterized by the presence of millimetric centrilobular nodularities with multiple linear ramifications [83]. The first description of tree-in-bud appearance was made in 1993 by Im et al. in CT scans of patients with active tuberculosis [84]; however, the tree-in-bud pattern—due to the dilation and filling of the terminal bronchioles by fluids, mucus, or pus—is a sign that can be found in many lung diseases [85]. The tree-in-bud appearance may occur in case of distal airway diseases, in bacterial, viral, and fungal infections, in some congenital diseases (for example cystic fibrosis), in some idiopathic diseases (bronchiolitis obliterans), in cases of inhalation/aspiration, in immune disorders, in some connectivitis, and in tumors that may cause centrilobular arterioles embolization. Nodular opacities with tree-in-bud appearance can be associated with other changes in lung parenchyma—such as thickening of the bronchial walls, consolidations, and/or areas of increased density with ground glass appearance [85]. In some cases, these opacities are associated with bronchiectasis and can reproduce a condition of small airways obstruction, with a mosaic-like appearance (air trapping).

### Tram track sign

**Fig. 34:**
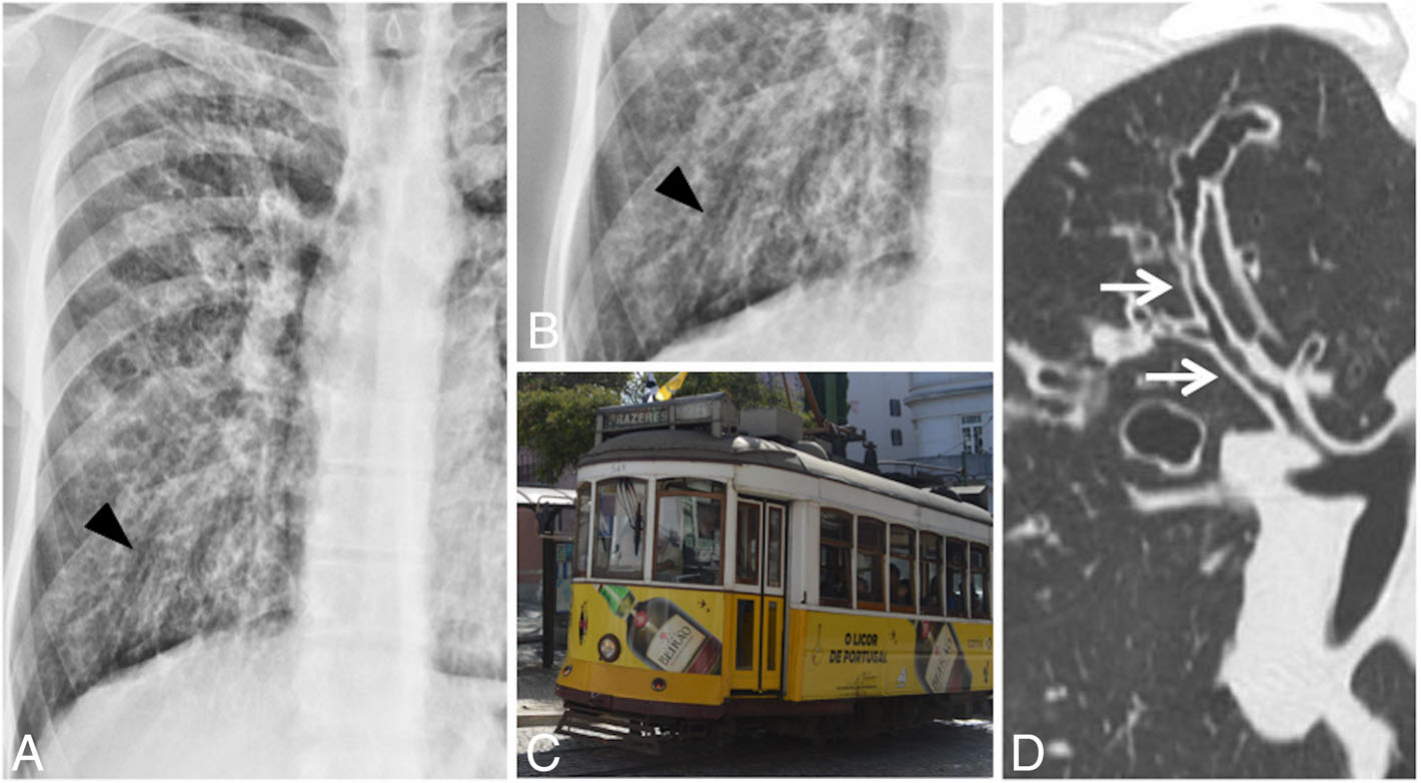
Tram track sign. The tram track sign may be explained by the presence of thickened bronchial branches on radiographs (black arrowheads in **a** and **b**), which reproduce a “tram line” appearance clearly shown on **c**. In **d**, the same appearance could be also described in a CT of a patient with cystic fibrosis: the coronal CT image shows thickened bronchial branches (cylindrical bronchiectasis)

In chest radiographs of patients affected by cylindrical bronchiectasis, the tram track sign is reproduced by the presence of thickened bronchial branches—which may reproduce a “tram line” appearance (Fig. 34). More in detail, bronchiectases are shown as parallel line opacities on chest radiography. The tram track sign can be more accurately appreciated on CT scans—where the pathological bronchus with thickened walls is well depicted in its major axis [86]. This “tram line” appearance is very frequently found in patients affected by cystic fibrosis with pulmonary involvement and in patients with COPD (chronic obstructive pulmonary disease) with severe bronchiectasis.

### Miscellaneous

Several other diseases or abnormalities may produce signs, symbols, and naturalistic figures: the serpent sign, the water lily sign, the cervicothoracic sign, the hilum overlay sign, the deep sulcus sign, the Hampton’s hump, the swiss-cheese sign, etc.

Among these radiological signs, two radiological features have been specifically described for pulmonary hydatid disease: they include the “serpent sign” and the “water lily sign.” In this regard, knowledge of these imaging appearances is very important for radiologists, considering that after hepatic localization the lung has been reported as the second most frequent site of disease for hydatid infection [87, 88]. The “water lily sign” is a pathognomonic radiological sign of hydatid infections; it is due to the detachment from the pericyst of the endocyst membrane, which collapses and fluctuates in the hydatid fluid—reproducing the appearance of a water lily [89]`. The serpent sign refers to the internal rupture of the hydatid cyst—with collapse of parasitic membranes into the cyst [90].

Other signs are very helpful for localization, such as the cervicothoracic sign or the hilum overlay sign; both signs are based on the “silhouette principle.” The cervicothoracic sign is used to differentiate if a mass—within the superior mediastinum—is located anteriorly or posteriorly, whereas the hilum overlay sign allows us to localize an opacity within the hilum, either anterior or posterior to it [6].

The deep sulcus sign refers to the radiolucency of the cardiophrenic or costophrenic angles in a supine chest X-ray, which is reproduced by the presence of a small air collection caused by a pneumothorax [91]. The Hampton’s hump sign is represented by a small peripheral consolidation, with triangular appearance, due to lung atelectasis in case of pulmonary emboli [92].

Finally, the so called “Swiss cheese sign” is used to indicate the appearance that the lung develops with pulmonary tears and pneumatoceles—filled with air and/or fluid after traumatic events [93]. The pneumatocele appears as a “hole” within the lung parenchyma that looks like Swiss cheese. In addition, other causes of pneumatocele include iatrogenic ventilation-induced pneumatocele (typical of newborns) and post-infectious diseases; this finding has been also described in ARDS [94].

## Conclusion

Several radiological features have been associated with common images, signs, symbols, and naturalistic figures—which may be encountered in everyday life. Linking specific imaging patterns to these symbols or naturalistic images, is easy to understand and remember, helps radiologists and non-radiologists in learning, and facilitates radiological recognition of various diseases. Although initially described as characteristic of a specific disease, many signs have been found in other pathological conditions; knowledge of the main differential and possible diagnoses—for each sign—is crucial for correct disease identification.

## Data Availability

Not applicable.

## Abbreviations

ARDS: Acute respiratory distress syndrome
BOOP: Bronchiolitis obliterans organizing pneumonia
CHP: Chronic hypersensitivity pneumonitis
COP: Cryptogenic organizing pneumonia
COPD: Chronic obstructive pulmonary disease
CT: Computed tomography
MDCT: Multidetector computed tomography
MPR: Multiplanar reformation
NSIP: Nonspecific interstitial pneumonia
UIP: Usual interstitial pneumonia

## References

[1] Hansell DM, Bankier AA, MacMahon H, McLoud TC, Müller NL, Remy J (2008). Fleischner Society: glossary of terms for thoracic imaging. Radiology.

[2] Tuddenham WJ (1984). Glossary of terms for thoracic radiology: recommendations of the Nomenclature Committee of the Fleischner Society. AJR Am J Roentgenol.

[3] Austin JH, Müller NL, Friedman PJ et al. (1996) Glossary of terms for CT of the lungs: recommendations of the Nomenclature Committee of the Fleischner Society. Radiology 200(2):327–331. 10.1148/radiology.200.2.8685321 10.1148/radiology.200.2.8685321 8685321

[4] Rémy J (1969). Silhouette sign and air bronchogram. Presse Med.

[5] Nawaz Khan A, Al-Jahdali H, Al-Ghanem S, Gouda A (2009). Reading chest radiographs in the critically ill (part II): radiography of lung pathologies common in the ICU patient. Ann Thorac Med.

[6] Algin O, Gökalp G, Topal U (2011). Signs in chest imaging. Diagn Interv Radiol.

[7] Abramson S (2001). The air crescent sign. Radiology.

[8] Bard R, Hassani N (1975). Crescent sign in pulmonary hematoma. Respiration.

[9] Curtis AM, Smith GJ, Ravin CE (1979). Air crescent sign of invasive aspergillosis. Radiology.

[10] Thompson BH, Stanford W, Galvin JR, Kurihara Y (1995). Varied radiologic appearances of pulmonary aspergillosis. Radiographics.

[11] Yella LK, Krishnan P, Gillego V (2005). The air crescent sign: a clue to the etiology of chronic necrotizing pneumonia. Chest.

[12] Ujita M, Renzoni EA, Veeraraghavan S, Wells AU, Hansell DM (2004). Organizing pneumonia: perilobular pattern at thin-section CT. Radiology.

[13] Zompatori M, Poletti V, Battista G, Diegoli M (1999). Bronchiolitis obliterans with organizing pneumonia (BOOP), presenting as a ring-shaped opacity at HRCT (the atoll sign). A case report. Radiol Med.

[14] Voloudaki AE, Bouros DE, Froudarakis ME, Datseris GE, Apostolaki EG, Gourtsoyiannis NC (1996). Crescentic and ring-shaped opacities. CT features in two cases of bronchiolitis obliterans organizing pneumonia (BOOP). Acta Radiol.

[15] Marchiori E, Zanetti G, Meirelles GS, Escuissato DL, Souza AS, Hochhegger B (2010). The reversed halo sign on high- resolution CT in infectious and noninfectious pulmonary diseases. AJR Am J Roentgenol.

[16] Kim SJ, Lee KS, Ryu YH et al. (2003) Reversed halo sign on high-resolution CT of cryptogenic organizing pneumonia: diagnostic implications. AJR Am J Roentgenol 180(5):1251–1254. 10.2214/ajr.180.5.1801251 10.2214/ajr.180.5.1801251 12704033

[17] Godoy MC, Viswanathan C, Marchiori E et al. (2012) The reversed halo sign: update and differential diagnosis. Br J Radiol 85(1017):1226–1235. 10.1259/bjr/54532316 10.1259/bjr/54532316 PMC3487053 22553298

[18] Legouge C, Caillot D, Chrétien ML et al. (2014) The reversed halo sign: pathognomonic pattern of pulmonary mucormycosis in leukemic patients with neutropenia? Clin Infect Dis 58(5):672–678. 10.1093/cid/cit929 10.1093/cid/cit929 24352351

[19] Marchiori E, Zanetti G, Irion KL et al. (2011) Reversed halo sign in active pulmonary tuberculosis: criteria for differentiation from cryptogenic organizing pneumonia. AJR Am J Roentgenol 197(6):1324–1327. 10.2214/AJR.11.6543 10.2214/AJR.11.6543 22109285

[20] Marchiori E, Zanetti G, Hochhegger B (2015). Reversed halo sign. J Bras Pneumol.

[21] Reed SL, O'Neil KM (1993). Cheerios in the chest. Chest.

[22] Lee KS, Kim Y, Han J, Ko EJ, Park CK, Primack SL (1997). Bronchiolalveolar carcinoma: clinical, histophatologic, and radiologic findings. Radiographics.

[23] Chou SH, Kicska G, Kanne JP, Pipavath S (2013). Cheerio sign. J Thoracic Imaging.

[24] Lee CH (2007). The crazy-paving sign. Radiology.

[25] Maimond N, Heimer D (2010). The crazy paving pattern on computed tomography. CMAJ.

[26] Rossi SE, Erasmus JJ, Volpacchio M, Franquet T, Castiglioni T, McAdams HP (2003). “Crazy-Paving” pattern at thin-section CT of the lungs: radiologic-pathologic overview. Radiographics.

[27] Verschakelen JA, Baert AL, Demedts M, Van Dyck H (1986). Rounded atelectasis of the lung: diagnosis on conventional radiology and CT. Eur J Radiol.

[28] Partap VA (1999). The comet tail sign. Radiology.

[29] Yadav P, Yadav PSeith A, Sood R (2006). The ‘dark bronchus’ sign: HRCT diagnosis of Pneumocystis carinii pneumonia. Ann Thorac Med.

[30] Mahomed N, Hleza B, Andronikou S (2011) The doughnut sign. S Aafr J Child Health 5(4):126–127

[31] Jacobson G, Felson B, Pendergrass EP, Flinn RH, Lainhart WS (1967). Eggshell calcifications in coal and metal miners. Semin Roentgenol.

[32] Gross BH, Schneider HJ, Proto AV (1980). Eggshell calcification of lymph nodes: an update. AJR Am J Roentgenol.

[33] Yudin A (2014). Feeding vessel sign or fruits on the branch sign. Metaphorical Signs in Computed Tomography of Chest and Abdomen.

[34] Dodd JD, Souza CA, Müller NL (2006). High-resolution MDCT of pulmonary septic embolism: evaluation of the feeding vessel sign. AJR Am J Roentgenol.

[35] Murata K, Takahashi M, Mori M et al. (1992) Pulmonary metastatic nodules: CT-pathologic correlation. Radiology 182(2):331–335. 10.1148/radiology.182.2.1732945 10.1148/radiology.182.2.1732945 1732945

[36] Nguyen ET (2003). The gloved finger sign. Radiology.

[37] Williams JR, Wilcox WC (1963) Pulmonary embolism: roentgenographic and angiographic considerations. AJR Am J Roentgenol Radium Ther Nucl Med 89:333–342 14000892

[38] Worsley DF, Alavi A, Aronchick JM, Chen JT, Greenspan RH, Ravin CE (1993) Chest radiographic findings in patients with acute pulmonary embolism: observations from the PIOPED Study. Radiology 1993;189(1):133–136. doi:10.1148/radiology.189.1.8372182 10.1148/radiology.189.1.8372182 8372182

[39] Fleischner FG (1961). Roentgen diagnosis of pulmonary embolism. Heart Bull.

[40] Kumaresh A, Kumar M, Dev B, Gorantla R, Sai PV, Thanasekaraan V (2015) Back to basics - 'must know' classical signs in thoracic radiology. J Clin Imaging Sci 31(5):43. 10.4103/2156-7514.161977 10.4103/2156-7514.161977 PMC4541161 26312141

[41] Nakatsu M, Hatabu H, Morikawa K et al. (2002) Large coalescent parenchymal nodules in pulmonary sarcoidosis: “sarcoid galaxy” sign. AJR Am J Roentgenol 178(6):1389–1393. 10.2214/ajr.178.6.1781389 10.2214/ajr.178.6.1781389 12034602

[42] Heo JN, Choi YW, Jeon SC, Park CK (2005). Pulmonary tubercolosis: another disease showing clusters of small nodules. AJR Am J Roentgenol.

[43] Ho ML, Gutierrez FR (2009). Chest radiography in thoracic polytrauma. AJR Am J Roentgenol.

[44] Warner R, Abbasi B et al. “Gingko leaf sign”. Available via https://radiopaedia.org/articles/ginkgo-leaf-sign-subcutaneous-emphysema. Accessed 20 Nov 2019.

[45] Golden R (1925) The effect of bronchostenosis upon the roentgen ray shadow in carcinoma of the bronchus. AJR Am J Roentgenol 13:21

[46] Lal Bunkar ML, Takhar R, Arya S, Biswas R (2014). Golden ‘S’ sign. BMJ Case Rep.

[47] Reinig JW, Ross P (1984). Computed tomography appearance of Golden’s “S” sign. J Comput Tomogr.

[48] Gupta P (2004). The Golden S sign. Radiology.

[49] Kuhlman JE, Fishman EK, Burch PA, Karp JE, Zerhouni EA, Siegelman SS (1988). CT of invasive pulmonary aspergillosis. AJR Am J Roentgenol.

[50] Lee YR, Choi YW, Lee KJ, Jeon SC, Park CK, Heo JN (2005). CT halo sign: the spectrum of pulmonary diseases. Br J Radiol.

[51] Alves GR, Marchiori E, Irion K et al. (2016) The halo sign: HRCT findings in 85 patients. J Bras Pneumol 42(6):435–439. 10.1590/S1806-37562015000000029 10.1590/S1806-37562015000000029 PMC5344092 28117474

[52] Parrón M, Torres I, Pardo M, Morales C, Navarro M, Martínez-Schmizcraft M (2008) The halo sign in computed tomography images: differential diagnosis and correlation with pathology findings. Arch Bronconeumol 2008;44(7):386–392. doi:10.1016/S0300-2896(08)70453-8 18727892

[53] Patel RA, Sellami D, Gotway MB, Golden JA, Webb WR (2000). Hypersensitivity pneumonitis: patterns on high-resolution CT. J Comput Assist Tomogr.

[54] Chong BJ, Kanne JP, Chung JH (2014). Headcheese sign. J Thorac Imaging.

[55] Oswald N, Parkinson T (1949). Honeycomb lungs. Q J Med.

[56] Lynch DA, Sverzellati N, Travis WD et al. (2018) Diagnostic criteria for idiopathic pulmonary fibrosis: a Fleischner Society White Paper. Lancet Respir Med 6(2):138–153. 10.1016/S2213-2600(17)30433-2 10.1016/S2213-2600(17)30433-2 29154106

[57] Naidich DP, Müller NL, Webb WR, Vlahos I, Krinsky GA (2007) Pattern of abnormality on high-resolution computed tomography. In: Naidich DP, Müller NL, Webb WR, Vlahos I, Krinsky GA (eds) Computed tomography and magnetic resonance of the thorax 4th edn. Lippincott Williams & Wilkins, Philadelphia.

[58] Sverzellati N, Lynch DA, Hansell DM, Johkoh T, King TE, Travis WD (2015). American Thoracic Society-European Respiratory Society Classification Idiopathic Interstitial Pneumonias: Advances in Knowledge since 2002. Radiographics.

[59] Pesle G, Monod O (1954). Bronchiectasis due to aspergilloma. Dis Chest.

[60] Nitschke A, Sachs P, Suby-Long T, Restauri N (2013). Monod sign. J Thorac Imaging.

[61] Sharma S, Dubey SK, Kumar N, Sundriyal D (2013) Monod sign and air crescent sign in aspergilloma. BMJ Case Rep. 10.1136/bcr-2013-200936 10.1136/bcr-2013-200936 PMC3794289 24038294

[62] Eber C, Stark P, Bertozzi P (1993) Bronchiolitis obliterans on high-resolution CT: a pattern of mosaic oligoemia. J Comput Assist Tomogr 17(6):853–856 10.1097/00004728-199311000-00003 8227568

[63] King MB, Harmon KR (1994). Unusual forms of pulmonary embolism. Clin Chest Med.

[64] Ridge CA, Bankier AA, Eisenberg RL (2011). Mosaic attenuation. AJR Am J Roentgenol.

[65] Stern EJ, Swensen SJ, Hartman TE, Frank MS (1995). CT mosaic pattern of lung attenuation; distinguishing different causes. AJR Am J Roentgenol.

[66] Li I, Greenstein J, Hahn B (2017). Pericardial effusion with oreo cookie sign. J Emerg Med.

[67] Wittram C, Maher MM, Yoo AJ, Kalra MK, Shepard JA, McLoud TC (2004). CT angiography of pulmonary embolism: diagnostic criteria and causes of misdiagnosis. Radiographics.

[68] Briccoli A, Farinetti A, Del Prete P, Rizzente AG, Saviano MS, Guernelli N (1993). Pulmonary hamartoma. Minerva Chir.

[69] Khan AN, Al-Jahdali HH, Allen CM, Irion KL, Al Ghanem S, Koteyar SS (2010). The calcified lung nodule: what does it mean?. Ann Thorac Med.

[70] Park CM, Goo JM (2009). Images in clinical medicine. “Popcorn” calcifications in a pulmonary chondroid hamartoma. N Engl J Med.

[71] Naidich DP, Sussman R, Kutcher WL, Aranda CP, Garay SM, Ettenger NA (1988). Solitary pulmonary nodules. CT-bronchoscopic correlation. Chest.

[72] Singh SP (1998). The positive bronchus sign. Radiology.

[73] Ernst A, Anantham D (2010). Bronchus sign on CT scan rediscovered. Chest.

[74] Tsuboi E, Ikeda S, Tajima M, Shimosato Y, Ishikawa S (1967). Transbronchial biopsy smear for diagnosis of peripheral pulmonary nodules and masses. Cancer.

[75] Robicsek F, Sanger PW, Taylor FH (1962). The diagnosis and treatment of the “scimitar syndrome”. Anomalous venous drainage of the right lung into the vena cava inferior. Coll Works Cardiopulm Dis.

[76] Nazarian J, Kanne JP, Rajiah P (2013). Scimitar sign. J Thorac Imaging.

[77] Ouellette H (1999). The signet ring sign. Radiology.

[78] Yudin A (2014). Snowstorm sign and cannonball metastases. Metaphorical signs in computed tomography of chest and abdomen.

[79] Al-Thuhli H, Al-Futaisi A (2007). Snow storm pattern. Oman Med J.

[80] Bhalotra B, Gogia A, Gupta P, Jain N (2004). A pulmonary snowstorm. Med J Aust.

[81] O’donovan PB (1997). The radiologic appearance of lung cancer. Oncology (Williston Park).

[82] Choromanska A, Macura KJ (2012). Evaluation of solitary pulmonary nodule detected during computed tomography examination. Pol J Radiol.

[83] Rossi SE, Franquet T, Volpacchio M, Giménez A, Aguilar G (2005). Tree-in-bud pattern at thin-section CT of the lungs: radiologic-pathologic overview. Radiographics.

[84] Im JG, Itoh H, Shim YS et al. (1993) Pulmonary tuberculosis: CT findings--early active disease and sequential change with antituberculous therapy. Radiology 186(3):653–660. 10.1148/radiology.186.3.8430169 10.1148/radiology.186.3.8430169 8430169

[85] Miller WT, Panosian JS (2013). Causes and imaging patterns of tree-in-bud opacities. Chest.

[86] Cantin L, Bankier AA, Eisenberg RL (2009). Bronchiectasis. AJR Am J Roentgenol.

[87] Sarkar M, Pathania R, Jhobta A, Thakur BR, Chopra R (2016). Cystic pulmonary hydatidosis. Lung India.

[88] Garg MK, Sharma M, Gulati A et al. (2016) Imaging in pulmonary hydatid cysts. World J Radiol 8(6):581–587. 10.4329/wjr.v8.i6.581 10.4329/wjr.v8.i6.581 PMC4919757 27358685

[89] Kaya HE, Kerimoğlu Ü (2017). The water lily sign. Abdom Radiol (NY).

[90] Kumar M, Raghavendra D, Mishra K, Suri V, Kumari S (2019). Pulmonary hydatid disease and serpent sign. QJM.

[91] Kong A (2003). The deep sulcus sign. Radiology.

[92] Reading M (1995). Chest X-ray quiz: Hampton’s hump. Aust Crit Care.

[93] Stark P, Greene R, Kott MM, Hall T, Vanderslice L (1987). CT findings in ARDS. Radiologe.

[94] Oikonomou A, Prassopoulos P (2011). CT imaging of blunt chest trauma. Insights Imaging.

